# A meta-analysis of genome-wide association studies of epigenetic age acceleration

**DOI:** 10.1371/journal.pgen.1008104

**Published:** 2019-11-18

**Authors:** Jude Gibson, Tom C. Russ, Toni-Kim Clarke, David M. Howard, Robert F. Hillary, Kathryn L. Evans, Rosie M. Walker, Mairead L. Bermingham, Stewart W. Morris, Archie Campbell, Caroline Hayward, Alison D. Murray, David J. Porteous, Steve Horvath, Ake T. Lu, Andrew M. McIntosh, Heather C. Whalley, Riccardo E. Marioni

**Affiliations:** 1 Division of Psychiatry, Centre for Clinical Brain Sciences, University of Edinburgh, Edinburgh, United Kingdom; 2 Centre for Dementia Prevention, University of Edinburgh, Edinburgh, United Kingdom; 3 Alzheimer Scotland Dementia Research Centre, University of Edinburgh, Edinburgh, United Kingdom; 4 Centre for Cognitive Ageing & Cognitive Epidemiology, University of Edinburgh, Edinburgh, United Kingdom; 5 Centre for Genomic and Experimental Medicine, Institute of Genetics and Molecular Medicine, University of Edinburgh, Edinburgh, United Kingdom; 6 Usher Institute for Population Health Sciences and Informatics, University of Edinburgh, Edinburgh, United Kingdom; 7 MRC Human Genetics Unit, Institute of Genetics and Molecular Medicine, University of Edinburgh, Edinburgh, United Kingdom; 8 Aberdeen Biomedical Imaging Centre, University of Aberdeen, Aberdeen, United Kingdom; 9 Department of Human Genetics, David Geffen School of Medicine, Los Angeles, CA, United States of America; 10 Department of Biostatistics, School of Public Health, University of California-Los Angeles, Los Angeles, CA, United States of America; Albert Einstein College of Medicine, UNITED STATES

## Abstract

'Epigenetic age acceleration' is a valuable biomarker of ageing, predictive of morbidity and mortality, but for which the underlying biological mechanisms are not well established. Two commonly used measures, derived from DNA methylation, are Horvath-based (Horvath-EAA) and Hannum-based (Hannum-EAA) epigenetic age acceleration. We conducted genome-wide association studies of Horvath-EAA and Hannum-EAA in 13,493 unrelated individuals of European ancestry, to elucidate genetic determinants of differential epigenetic ageing. We identified ten independent SNPs associated with Horvath-EAA, five of which are novel. We also report 21 Horvath-EAA-associated genes including several involved in metabolism (*NHLRC*, *TPMT*) and immune system pathways (*TRIM59*, *EDARADD*). GWAS of Hannum-EAA identified one associated variant (rs1005277), and implicated 12 genes including several involved in innate immune system pathways (*UBE2D3*, *MANBA*, *TRIM46*), with metabolic functions (*UBE2D3*, *MANBA*), or linked to lifespan regulation (*CISD2*). Both measures had nominal inverse genetic correlations with father’s age at death, a rough proxy for lifespan. Nominally significant genetic correlations between Hannum-EAA and lifestyle factors including smoking behaviours and education support the hypothesis that Hannum-based epigenetic ageing is sensitive to variations in environment, whereas Horvath-EAA is a more stable cellular ageing process. We identified novel SNPs and genes associated with epigenetic age acceleration, and highlighted differences in the genetic architecture of Horvath-based and Hannum-based epigenetic ageing measures. Understanding the biological mechanisms underlying individual differences in the rate of epigenetic ageing could help explain different trajectories of age-related decline.

## Author summary

DNA methylation, an epigenetic process, is known to vary with age. Methylation levels at specific sites across the genome can be combined to form estimates of age known as ‘epigenetic age’. The difference between epigenetic age and chronological age is referred to as ‘epigenetic age acceleration’, with positive values indicating that a person is biologically older than their years. Understanding why some people seem to age faster than others could shed light on the biological processes behind age-related decline; however, the mechanisms underlying differential rates of epigenetic ageing are largely unknown. Here, we investigate genetic determinants of two commonly used epigenetic age acceleration measures, based on the Horvath and Hannum epigenetic clocks. We report novel genetic variants and genes associated with epigenetic age acceleration, and highlight differences in the genetic factors influencing these two measures. We identify ten genetic variants and 21 genes associated with Horvath-based epigenetic age acceleration, and one variant and 12 genes associated with the Hannum-based measure. There were no genome-wide significant variants or genes in common between the Horvath-based and Hannum-based measures, supporting the hypothesis that they represent different aspects of ageing. Our results suggest a partial genetic basis underlying some previously reported phenotypic associations.

## Introduction

Ageing is associated with a decline in physical and cognitive health, and is the main risk factor for many debilitating and life-threatening conditions including cardiovascular disease, cancer, and neurodegeneration [1]. Ageing is a multi-dimensional construct, incorporating physical, psychosocial, and biological changes. Everyone experiences the same rate of chronological ageing, but the rate of ‘biological ageing’, age-related decline in physiological functions and tissues, differs between individuals. Various phenotypic and molecular biomarkers have been used to study biological ageing, including a number of 'biological clocks', the best known of which is telomere length. Telomeres shorten with increasing age, and telomere length has been found to predict morbidity and mortality [2]. More recently, research into epigenetics–chemical modifications to DNA without altering the genetic sequence–has yielded another method for measuring biological age.

DNA methylation is an epigenetic modification, typically characterised by the addition of a methyl group to a cytosine-guanine dinucleotide (CpG) [3], that can influence gene expression and is associated with variation in complex phenotypes. This process is essential for normal development and is associated with a number of key processes including ageing. DNA methylation levels are dynamic, varying with age across the life course [4,5] and are influenced by both genetic and environmental factors [6].

Weighted averages of methylation at multiple CpG sites can be integrated into estimates of chronological age referred to as ‘epigenetic age’. Two influential studies have used this method to create ‘epigenetic clocks’, which accurately predict chronological age in humans. Hannum et al. used DNA methylation profiles from whole blood from two cohorts to identify 71 CpG sites that could be used to generate an estimate of age [7], while Horvath used data from 51 different tissue types from multiple studies to identify 353 CpG sites whose methylation levels can be combined to form an age predictor [8]. Hannum et al.’s clock is specific to blood samples, although it can be adjusted for different tissue types using linear models. The Horvath clock is widely applicable, with the same CpG set and the same algorithm being used irrespective of the DNA source.

Although similar penalised regression models were used to select the CpG sites to be included in each of these epigenetic clocks, there is limited overlap in the CpGs included. The two measures are clearly related, but are thought to capture slightly different aspects of the biology of ageing [9]. The Hannum age estimator correlates with proportions of certain blood cells, reflecting its construction based on blood methylation data [9,10], and it is considered to track aspects of immunosenescence. The pan-tissue Horvath clock, constructed across a broad spectrum of tissue and cell types, is relatively uncorrelated with blood cell proportions [11], and is thought to capture cell-intrinsic changes in DNA methylation which might reflect an innate ageing process.

Both the Hannum and Horvath epigenetic clocks are strongly correlated (r>0.95) with chronological age [7,8]. However, despite these high overall correlations, there can be substantial differences between epigenetic and chronological age at the individual level, and it is unclear what drives these differences. A greater epigenetic age relative to chronological age is commonly described as ‘epigenetic age acceleration’ (EAA), and implies that a person is biologically older than their years. EAA has been shown to be informative for both current and future health trajectories [9]. Recently, a growing number of studies have used EAA to investigate age-related disorders, and the epigenetic clock is increasingly being recognised as a valuable marker of biological ageing [10,12].

The simplest definition of epigenetic age acceleration is the residual that results from regressing epigenetic age on chronological age. However, it is well known that the abundance of different cell types in the blood changes with age [13,14], and hence two broad categories of EAA measures have been distinguished: those that are independent of age-related changes in blood cell composition, and those that incorporate and are enhanced by blood cell count information [10]. The former group, considered to reflect ‘pure’ epigenetic ageing effects that are not influenced by differences in blood cell counts, are often referred to as ‘intrinsic’ epigenetic age measures. The latter group up-weights the contributions of blood cell counts, thus leveraging known age-related changes to blood cell proportions to capture aspects of immunosenescence; these measures are referred to as ‘extrinsic’ epigenetic age measures.

In keeping with previous work, this study focuses on two different epigenetic age measures, based on the Horvath and Hannum epigenetic clocks [7,8], and uses these to derive variations of EAA that are either independent of blood cell counts, or enhanced by changes in blood cell composition. Horvath-based epigenetic age follows the approach by Horvath (2013), and is defined as the predicted value of age based on the DNA methylation levels of the 353 CpG sites identified in his study [8]. Horvath-based epigenetic age acceleration (Horvath-EAA) is the residual term of a multivariate model regressing the Horvath-based epigenetic age estimate on chronological age and estimates of blood cell counts. It is by definition independent of both chronological age and age-related changes in the cellular composition of blood. Hannum-based epigenetic age is based on DNA methylation levels at the 71 CpGs identified by Hannum et al. (2013) [7]. Hannum-based epigenetic age acceleration (Hannum-EAA) is an enhanced version of the Hannum estimate which up-weights the contributions of age-associated blood cells. A weighted average of Hannum-based epigenetic age with blood cells whose abundance is known to change with age is calculated, and Hannum-EAA is then defined to be the residual variation from a univariate model regressing the weighted DNA methylation age estimate on chronological age. Hannum-EAA is independent of chronological age but in addition to cell-intrinsic epigenetic changes it also tracks age-related changes in blood cells. Full details of the calculation of Horvath-EAA and Hannum-EAA are given in **S1 Text**.

Horvath-EAA, described in previous publications as ‘intrinsic’ epigenetic age acceleration (IEAA), can be interpreted as a measure of cell-intrinsic ageing that exhibits preservation across multiple tissues, appears unrelated to lifestyle factors, and probably indicates a fundamental cell ageing process that is largely conserved across cell types [8,10]. In contrast, Hannum-EAA, referred to in previous studies as ‘extrinsic’ epigenetic age acceleration (EEAA), can be considered a biomarker of immune system ageing, explicitly incorporating aspects of immune system decline such as age-related changes in blood cell counts, correlating with lifestyle and health-span related characteristics, and thus yielding a stronger predictor of all-cause mortality [10,15].

It should be noted that as both the Horvath and Hannum epigenetic clocks correlate well with age, in a population with a wide age range they are guaranteed to correlate with each other. However, Horvath-based and Hannum-based epigenetic age acceleration estimates, i.e. the degree of divergence of epigenetic age from chronological age, are not guaranteed to be correlated.

Previous studies have identified relationships between epigenetic ageing and numerous traits, including several age-related health outcomes, for example Alzheimer’s disease pathology [16], cognitive impairment [16], and age at menopause [17]. Higher EAA has been associated with poorer measures of physical and cognitive fitness [9] and higher risk of all-cause mortality [12]. Many associations are specific to either Horvath-EAA or Hannum-EAA, a discordance that may reflect the differences in the two estimates and supports the theory that they represent different aspects of ageing [15,18,19].

While EAA has been associated with various markers of physical and mental fitness, the mechanisms underlying epigenetic ageing remain largely unknown. There has been little research conducted thus far on genetic contributions to epigenetic age acceleration. However, Lu et al. (2018) recently published results of the first genome-wide association analysis of blood EAA in a sample of 9,907 individuals, identifying five genetic loci associated with Horvath-EAA and three Hannum-EAA-associated loci [20].

This current study, with a sample size of 13,493 individuals, constitutes the largest study of the genetic determinants of DNA methylation-based ageing to date. Single nucleotide polymorphism (SNP)-based and gene-based approaches were used to identify genes and loci associated with Hannum-based and Horvath-based estimates of EAA. Functional mapping and annotation of genetic associations were performed, alongside gene-based and gene-set analyses, in an attempt to elucidate the genes and pathways implicated in differential rates of epigenetic ageing between individuals and shed light on the underlying biological mechanisms. We report novel SNPs and genes associated with epigenetic age acceleration, and highlight differences in the genetic architectures of the Horvath-based and Hannum-based EAA measures.

## Results

### Estimation of epigenetic age and epigenetic age acceleration in the Generation Scotland sample

**Fig 1:**
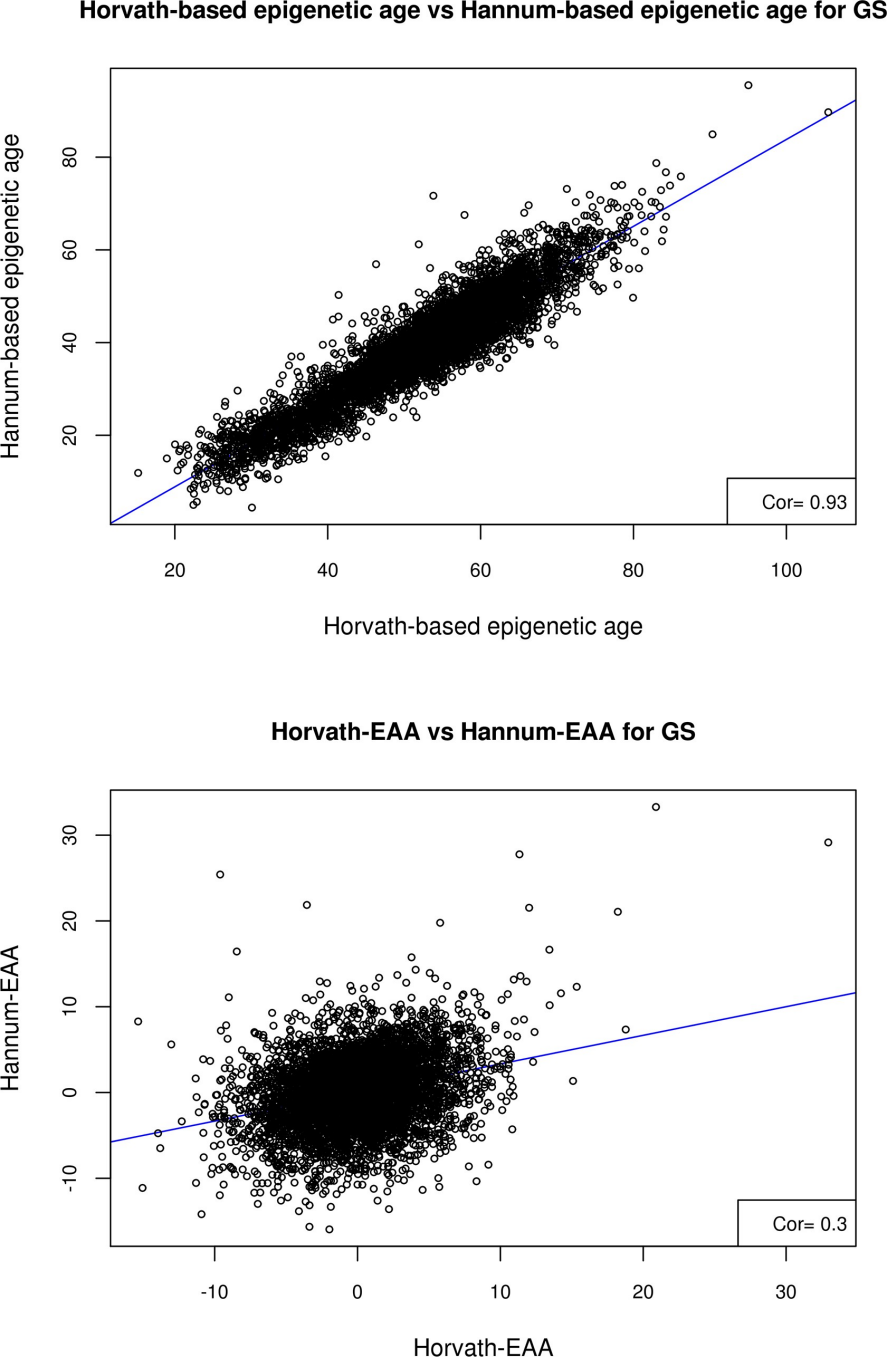
Scatter plots of A) Horvath-based epigenetic age versus Hannum-based epigenetic age, and B) Horvath-EAA vs Hannum-EAA, for the Generation Scotland sample.

A summary of the estimated epigenetic age variables in Generation Scotland (GS) is given in **S1 Table**. Both the Horvath- and Hannum-based estimates of biological age were highly correlated with chronological age (r = 0.94, SE = 0.005 and r = 0.93, SE = 0.005 respectively). The two DNA methylation age estimates were also highly correlated with each other (r = 0.93, SE = 0.005); however, the two estimates of epigenetic age acceleration, Horvath-EAA and Hannum-EAA, were only weakly correlated (r = 0.30, SE = 0.013) (**Fig 1**).

### GWAS of Horvath-EAA and Hannum-EAA in GS and replication of previously identified loci

**Fig 2:**
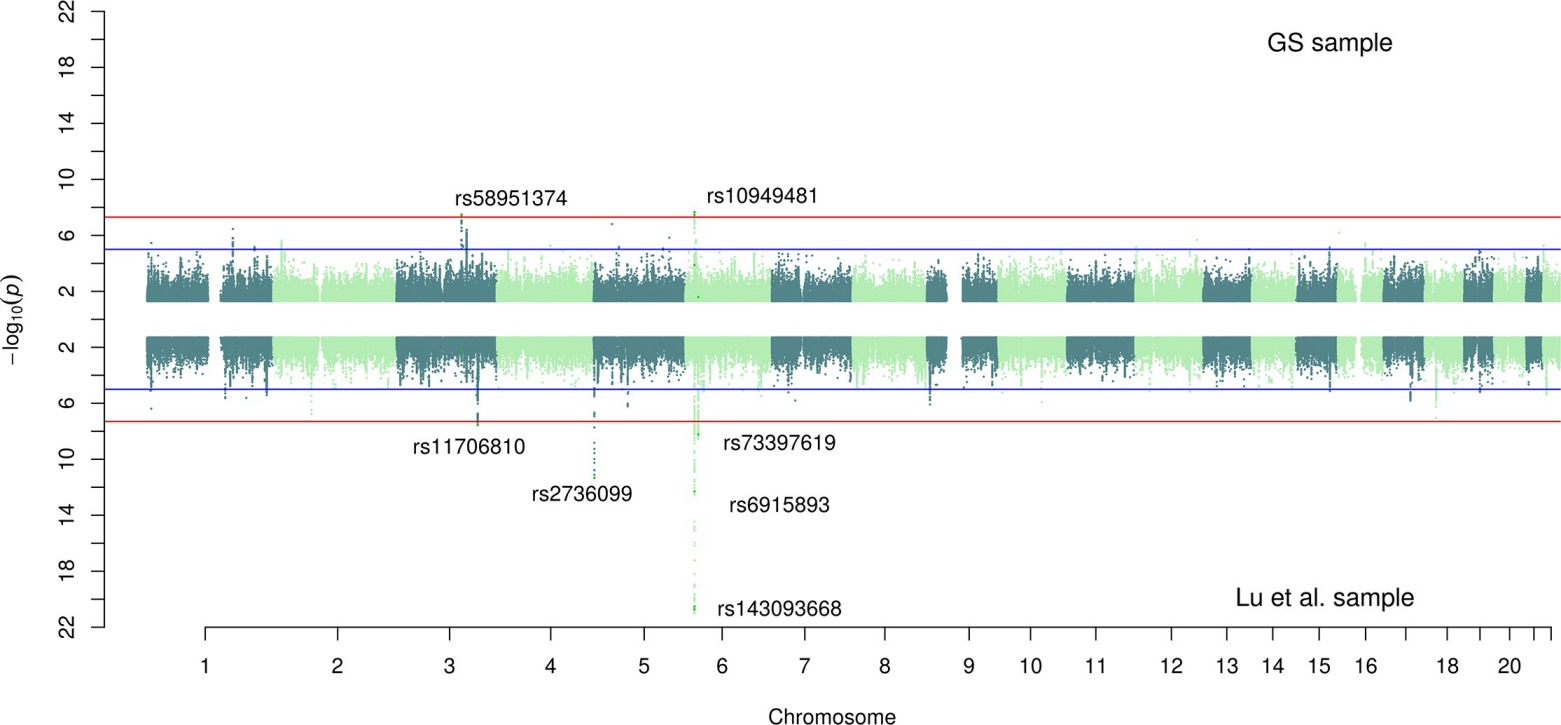
Miami plot for GWAS of Horvath-EAA in the GS and Lu et al. cohorts. SNP-based Miami plot comparing the results of genome-wide association analyses of Horvath-based epigenetic age acceleration in GS (top, n = 5,100) and Lu et al. (bottom, n = 8393), with—log_10_ transformed *P*-values for each SNP plotted against chromosomal location. The red line indicates the threshold for genome-wide significance (*P*<5×10^−8^) and the blue line for suggestive associations (*P*<1×10^−5^). Independent significant SNPs are annotated.

**Fig 3:**
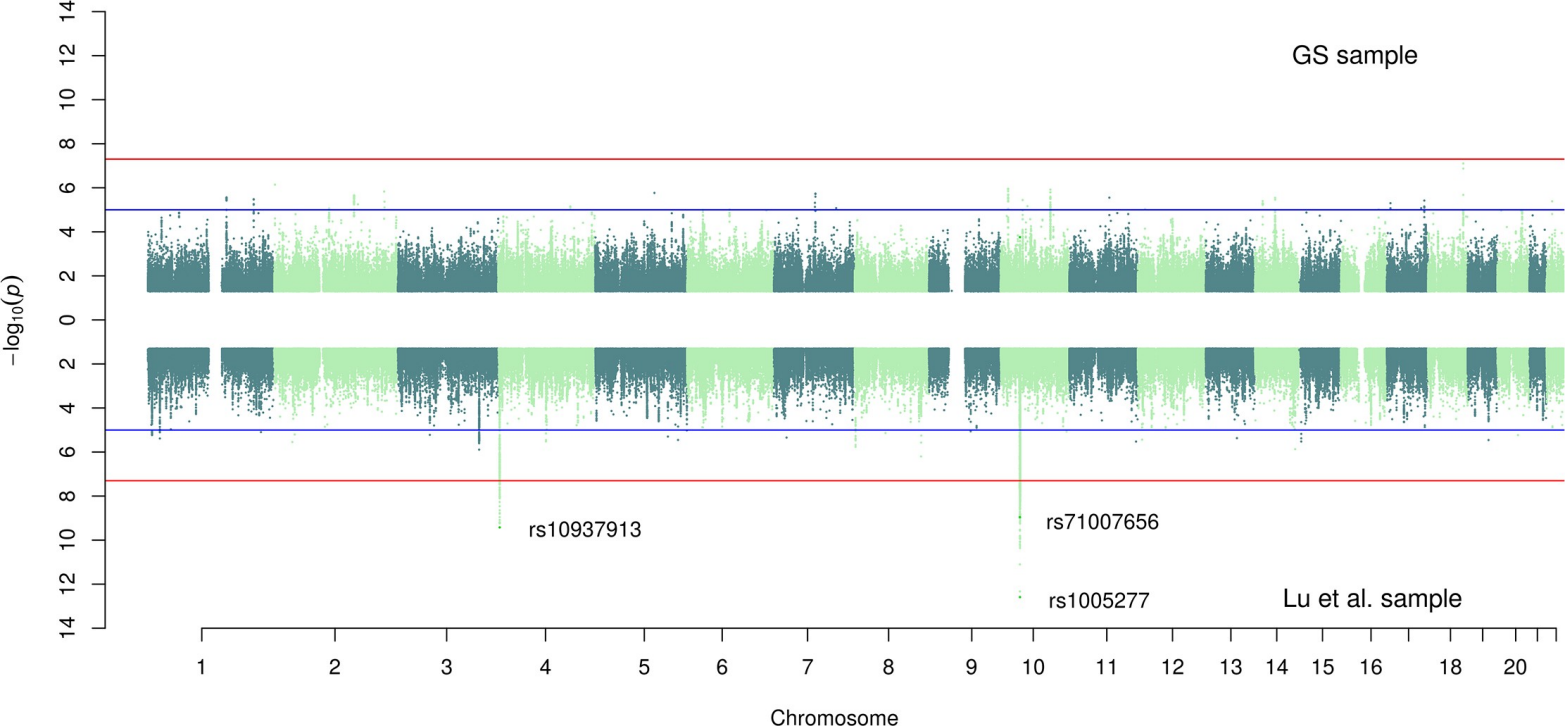
Miami plot for GWAS of Hannum-EAA in the GS and Lu et al. cohorts. SNP-based Miami plot comparing the results of genome-wide association analyses of Hannum-based epigenetic age acceleration in GS (top, n = 5,100) and Lu et al. (bottom, n = 8393), with—log_10_ transformed *P*-values for each SNP plotted against chromosomal location. The red line indicates the threshold for genome-wide significance (*P*<5×10^−8^) and the blue line for suggestive associations (*P*<1×10^−5^). Independent significant SNPs are annotated.

The genome-wide association study (GWAS) for the GS cohort yielded two significant (*P*<5x10^-8^) variants for Horvath-EAA, but no SNPs achieved genome-wide significance for association with Hannum-EAA (minimum *P*-value 7.85x10^-8^) (**S2 Table,** full output available online at https://doi.org/10.7488/ds/2631). There was a moderate genetic correlation between the two traits in the GS sample (r_G_ = 0.597, SE = 0.279), and both measures had high genetic correlations with the previously reported findings of Lu et al. (r_G_ = 0.724, SE = 0.312 and r_G_ = 1.021, SE = 0.356 for Horvath-EAA and Hannum-EAA respectively). All the significant SNPs from the Lu et al. analysis of Horvath-EAA had the same direction of effect in GS (**S3 Table**), with one attaining genome-wide significance (rs143093668, *P*-value = 3.53x10^-8^; remaining SNPs had *P*-values between 5.76x10^-2^ and 1.34x10^-4^). Two of the three significant SNPs from Lu et al.'s GWAS of Hannum-EAA had the same direction of effect in GS, although not at genome-wide significance levels in this smaller sample (*P-*values 1.76x10^-3^ and 1.75x10^-4^). Miami plots demonstrating a comparison between the EAA SNP association profiles in the GS and Lu et al. samples are shown in **Fig 2** (Horvath-EAA) and **Fig 3** (Hannum-EAA). Quantile-quantile plots (QQ plots) for the GWAS of Horvath-EAA and Hannum-EAA in GS are shown in **S1 Fig**.

### GWAS meta-analysis

**Fig 4:**
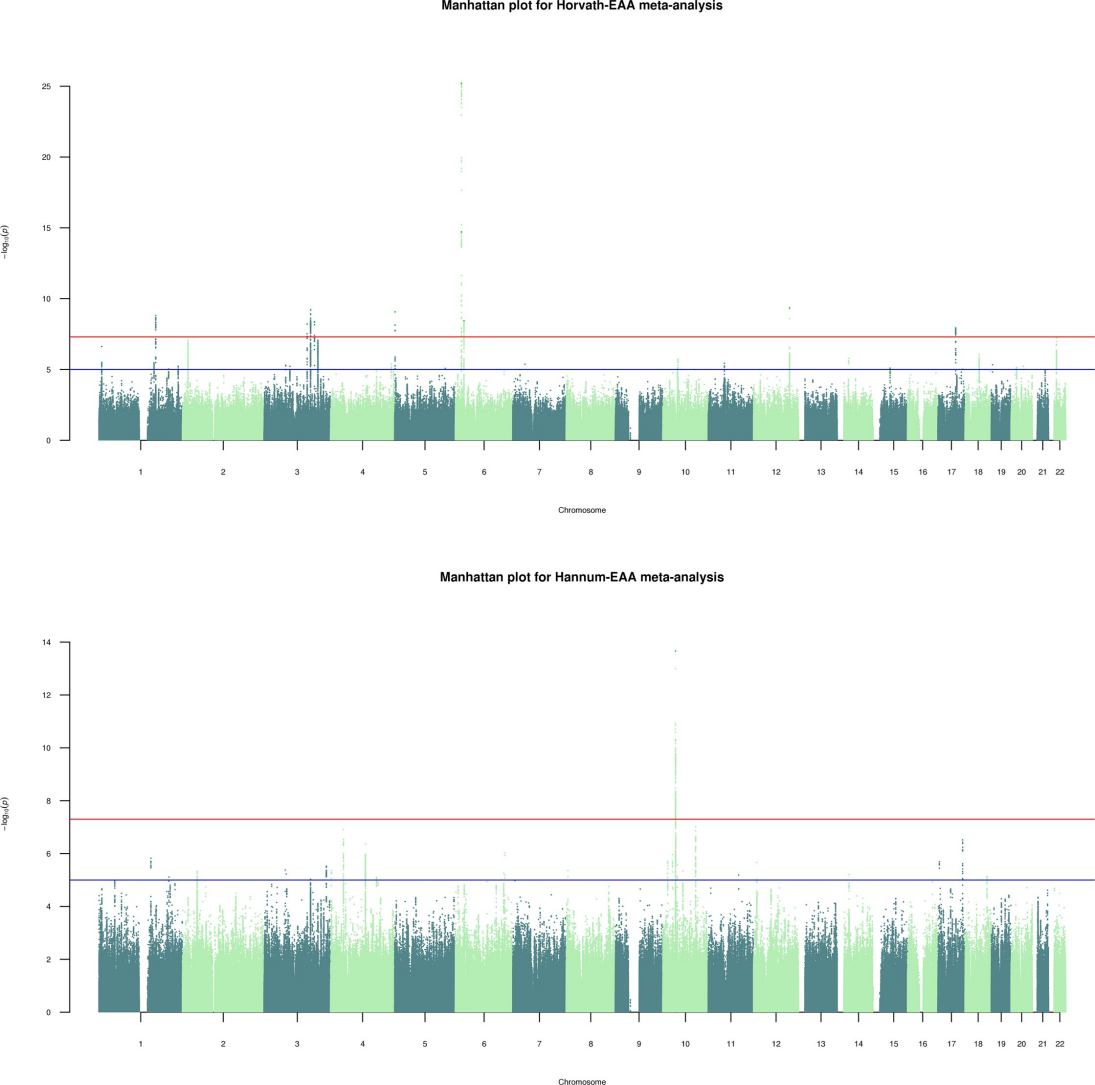
Manhattan plots for genome-wide meta-analyses (n = 13,493) of Horvath-based and Hannum-based epigenetic age acceleration. SNP-based Manhattan plots for Horvath-EAA and Hannum-EAA, with—log_10_ transformed *P*-values for each SNP plotted against chromosomal location. The red line indicates the threshold for genome-wide significance (*P*<5×10^−8^) and the blue line for suggestive associations (*P*<1×10^−5^). Independent significant variants are annotated.

**Fig 5:**
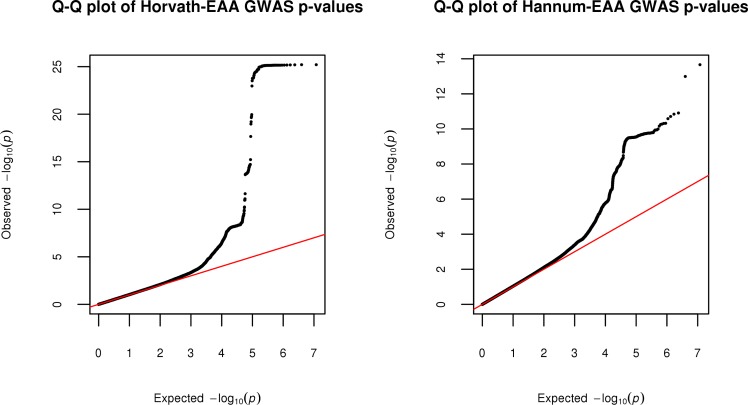
QQ plots for the meta-analyses of Horvath-based and Hannum-based epigenetic age acceleration. Quantile-quantile plots for the genome-wide meta-analyses of Horvath-EAA and Hannum-EAA, showing the expected distribution of GWAS test statistics, -log10(p), versus the observed distribution.

We conducted genome-wide association meta-analyses of Horvath-EAA and Hannum-EAA using 13,493 European-ancestry individuals aged between ten and 98 years from 12 cohorts, adjusting for sex. Manhattan plots for Horvath-EAA and Hannum-EAA are shown in **Fig 4**, with QQ plots of the observed *P*-values versus those expected shown in **Fig 5**. We did not find apparent evidence for genomic inflation in either the GS study (Horvath-EAA: genomic inflation factor λ_GC_ = 1.017, Linkage Disequilibrium (LD) score regression intercept (SE) = 1.002 (0.007); Hannum-EAA: λ_GC_ = 1.023, intercept (SE) = 0.998 (0.006), **S4 Table**) or the meta-analysis (Horvath-EAA: λ_GC_ = 1.035, intercept (SE) = 1.006 (0.008), Hannum-EAA: λ_GC_ = 1.044, intercept (SE) = 1.002 (0.007)); Lu et al. previously reported no evidence for genomic inflation for any of the individual studies making up their meta-analysis [20].

We identified 439 variants with a genome-wide significant association (*P*<5×10^−8^) with Horvath-EAA, of which ten were independent (*r*^2^<0.1 within a 250kb window). The significantly associated variants mapped to nine genomic loci on six chromosomes (**Table 1**, full details in **S5 Table**). Of the ten independent significant variants identified here, five were novel, that is, not within ± 500 Kb of a significant variant (*P*<5×10^−8^) reported by Lu et al. [20]. The novel findings were a SNP on chromosome 1q24.2 in the *C1orf112* gene, three SNPs on chromosome three, at 3q21.3 (nearest gene: *GATA2-AS1*), 3q22.3 in the *PIK3CB* gene, and 3q25.1 in the *LINC01214* gene, and a SNP on chromosome 12q23.3 (nearest genes: *RP11-412D9*.*4* and *TMEM263*). The risk alleles at these loci conferred between 0.33 (SE = 0.054) and 1.34 (SE = 0.127) years higher Horvath-EAA (**Table 1**). These ten independent lead SNPs showed complete sign concordance for association with Horvath-EAA across GS and the Lu study (**S6 Table**). Comparing the genomic loci identified in the current study with the five reported by Lu et al., only one locus that was previously reported was not identified at genome-wide significance here (rs11706810 at 3q25.33, meta-analysis *P*-value 8.68x10^-8^). **S2 Fig** shows the regional association plots for the independent signals, visualised in LocusZoom [21]. Of the ten independent SNPs achieving genome-wide significance, none associated with any other phenotype in currently published GWAS available via the NHGRI-EBI catalog.

**Table 1:** Independent variants with a meta-analysis genome-wide significant association with Horvath-based or Hannum-based epigenetic age acceleration.

Phenotype	Index SNP	Chromosome	Position	A1/A2	Freq	Beta	SE	*P*-value	Gene^a^	function	Previously reported
**Horvath-EAA**	rs1011267	1q24.2	169677720	A/G	0.503	-0.327	0.054	1.579E-09	*C1orf112*	intron variant	novel
	rs79070372	3q21.3	128510481	A/G	0.111	0.505	0.087	6.074E-09	*GATA2-AS1*	non coding transcript variant	novel
	rs388649	3q22.3	138777967	A/T	0.495	-0.338	0.055	6.054E-10	*PIK3CB*	intron variant	novel
	rs6440667	3q25.1	150287063	C/G	0.161	0.440	0.075	4.28E-09	*LINC01214*	intron variant	novel
	rs2736099	5p15.33	1287225	A/G	0.367	0.373	0.061	8.58E-10	*TERT*	intron variant	yes
	rs7744541	6p22.3	18104469	A/T	0.418	0.439	0.055	1.93E-15		intergenic variant	yes
	rs76244256	6p22.3	18140332	T/C	0.046	-1.341	0.127	6.231E-26	*TPMT*	intron variant	yes
	rs4712953	6p22.2	25671618	A/T	0.725	0.346	0.059	3.604E-09	*SCGN*	intron variant	yes
	rs10778517	12q23.3	106947886	T/G	0.565	0.335	0.054	4.46E-10	*RP11-412D9*.*4/TMEM263*	unknown	novel
	rs62078811	17q22	55031815	A/G	0.218	-0.369	0.065	1.158E-08	*STXBP4*	intron variant	yes
**Hannum-EAA**	rs1005277	10p11.21	37929331	A/C	0.301	0.533	0.070	2.173E-14		unknown	yes

Genome-wide significance defined as having a *P*-value of *P*<5x10^-8^. A1 and A2 refer to the reference allele and non-reference allele for the index SNP, respectively. Freq (allele frequency), Beta (effect size), and SE (standard error of effect size) columns pertain to the reference allele, A1. Chromosome and position (in Mb) denote the location of the index SNP, and are given with regards to the GRCh38 assembly.

a Genes are listed if located within +/- 10 kb of a listed SNP.

The Hannum-EAA GWAS meta-analysis identified 324 genome-wide significant (*P*<5×10^−8^) associated variants mapping to a single genomic locus at 10p11.21 with one index SNP (**Fig 4**, **Table 1**, full details of index SNP in **S5 Table**). *ZNF25*, a transcription factor associated with osteoblast differentiation of human skeletal stem cells [22], is the closest gene to this variant, at a distance of 20 Kb. At this Hannum-EAA-related locus, the risk allele conferred 0.53 (SE = 0.070) years higher Hannum-EAA. We replicated two of the three variants significantly associated with Hannum-EAA in the Lu et al. study; however, based on our clumping criteria with *r*^2^<0.1, we report only one as an independent significant SNP. Conditional analysis revealed no secondary signal at this locus. The third locus reported in the previous study was not associated at genome wide significance in this larger sample (*P =* 3.74x10^-3^). A regional association plot for 10p11.21 is shown in **S2J Fig**.

Of the ten independent variants associated with Horvath-EAA, nine exhibited sign-consistent associations with Hannum-EAA, of which five attained at least nominal significance with association *P*-values less than 0.05 (most significant *P* = 6.9x10^-5^) (**S7 Table**). The single independent SNP associated with Hannum-EAA also exhibited a nominal and sign-consistent association with Horvath-EAA (*P* = 0.011).

### Methylation quantitative trait loci

Multiple studies have found that individual genotypes at specific loci can influence patterns of DNA methylation (e.g. [23,24]). These loci, referred to as methylation quantitative trait loci (mQTL) can influence methylation across extended genomic regions [23,24], and may underlie some SNP-phenotype associations. To evaluate whether mQTL are driving the observed associations between SNPs and epigenetic age acceleration in our analysis, we assessed whether any of the independent significant SNPs from the Horvath-EAA and Hannum-EAA GWAS meta-analysis are mQTL for any CpGs included in the Horvath or Hannum epigenetic clocks, using the methylation quantitative trait loci database (mQTLdb, [25]).

The single Hannum-EAA genome-wide significant SNP, rs1005277, is an mQTL for 38 different CpGs across the five assessed time points (birth, childhood, adolescence, middle age, pregnancy). For 11 of these CpGs the mQTL is *cis*-acting (where the genetic variation occurs close to the methylation site), while it acts in *trans* (where variation occurs elsewhere in the genome) for the other 27 CpGs. None of these CpGs, however, are included in either the Horvath or Hannum epigenetic clocks.

Nine of the ten Horvath-EAA independent significant SNPs are mQTL, for a total of 74 different CpGs in the mQTL database. Two of these CpGs, cg26297688 and cg01459453, are included in the Horvath clock only, while one, cg22736354, intersects with both the Hannum and Horvath clocks. Four of the Horvath-EAA SNPs are *cis*-acting mQTL for these clock CpGs at multiple time points, with two SNPs acting as mQTL for the same CpG (**S8 Table**). These results suggest a potential mechanism of action whereby these SNPs influence biological ageing through their effect on methylation levels. A summary of the CpGs linked to each mQTL is shown in **S9 Table.**

### Heritability

In order to characterise the genetic contribution to accelerated epigenetic ageing, SNP-based heritability was estimated using univariate LD score regression [26], which requires only GWAS summary statistics rather than full genotype data. The SNP-based heritabilities of Horvath-EAA and Hannum-EAA were estimated to be 0.154 (SE = 0.042) and 0.194 (SE = 0.040) respectively (**S4 Table**), providing evidence for a genetic component to differential epigenetic ageing rates. These figures are comparable to previous SNP-based heritability estimates but lower than estimates based on pedigree relationships [20].

### SNP functional annotation

We used FUMA [27] to functionally annotate SNPs in LD (r^2^≥0.6) with the independent significant SNPs for each of the epigenetic age acceleration measures. For Horvath-EAA, this resulted in functional annotation of 825 SNPs (**S10 Table**). The vast majority of the SNPs were intergenic (44.85%) or intronic (47.88%), with only five (0.61%) exonic SNPs. 25 SNPs had CADD (Combined Annotation Dependent Depletion) scores greater than 12.37, surpassing the suggested threshold to be considered deleterious and thus providing evidence of pathogenicity [28]. The highest CADD scores were found in three exonic SNPs: rs1800460 and rs1142345 of *TPMT* and rs10949483 of *NHLRC1* (CADD scores 28.40, 28.30 and 18.92 respectively), indicating potentially deleterious protein effects. Six SNPs (rs413147, rs12631035, rs9851887, rs12189658, rs6915893, rs12199316) had RegulomeDB scores below 2, suggesting that variation at these SNPs is likely to affect gene expression [29]. Almost all SNPs (98.18%) were in open chromatin regions.

For Hannum-EAA, functional annotation of 1,382 candidate SNPs indicated a high proportion of intergenic SNPs (60.49%), while 11.79% were intronic and only three SNPs were located in exons (**S11 Table**). 14 SNPs had CADD scores above 12.37, indicating that variation at these SNPs is potentially deleterious. Although 42.04% of the SNPs were located in open chromatin regions, there is little evidence that the Hannum-EAA-associated locus contains regulatory regions, as analysis using RegulomeDB, which integrates a larger collection of regulatory information encompassing protein binding, motifs, expression quantitative trait loci (eQTL), and histone modifications as well as chromatin structure, revealed only one SNP (rs2474568) with a score below 2.

### eQTL and colocalisation analysis

For each independent SNP associated with Horvath-EAA or Hannum-EAA, evidence of eQTL was explored using the Genotype Tissue Expression (GTEx) v7 database [30]. Seven of the ten independent significantly associated SNPs for Horvath-EAA were identified as potential eQTL (**S12 Table**). Notably, rs388649 is associated with expression of *ESYT3*, which has a role in lipid transport and metabolism pathways [31,32], expression of *FAIM*, which is associated with apoptosis and autophagy [33], in a number of skin and brain tissues, and *PIK3CB*, which regulates vital cell functions including proliferation and survival [34,35]. rs76244256, the variant most strongly associated with Horvath-EAA, shows eQTL evidence for *NHLRC1* expression, which is associated with glycogen metabolism [36], across multiple tissues. We found no evidence for the Hannum-EAA-associated SNP, rs1005277, regulating gene expression.

To further investigate the possibility that these SNPs act via regulating the expression of genes, we carried out colocalisation analysis using a Bayesian statistical method implemented in the 'coloc' package in R [37], which uses an approximate Bayes factor to estimate the posterior probability (PP) that a given variant is causal in both the GWAS and eQTL studies. We integrated our GWAS data with *cis*-eQTL data from the eQTLGen Consortium (https://www.eqtlgen.org/) [38] and analysed pairwise colocalisation within a +/- 200 kb window of each significant SNP. These analyses provide no evidence that the effect of these SNPs on accelerated epigenetic ageing is mediated through *cis* gene expression. There was no evidence for colocalisation of any Horvath-EAA or Hannum-EAA-associated SNP with *cis*-eQTL (PP for shared causal variant 8.31x10^-15^–0.030, **S13 Table**). Rather, in all but one case, the results support the hypothesis that there are two distinct causal variants affecting epigenetic age acceleration and transcript levels in the region (PP>0.95). In the +/-200 kb region surrounding variant rs2736099, there is strong evidence (PP>0.95) for a causal variant affecting gene expression, but not EAA.

### Gene-based analysis

MAGMA (Multi-marker Analysis of GenoMic Annotation) v1.6 was used to identify gene-level associations with each EAA measure [39]. SNPs were mapped to 17,798 protein coding genes, with genome-wide significance defined at *P =* 0.05/17,798 = 2.809x10^-6^. A total of 21 genes attained genome-wide significance for association with Horvath-EAA (**Table 2**, full details in **S14 Table**). As expected, many of these genes were located in the same regions as the lead SNPs. Three genes at 6p22.3, *NHLRC1*, *TPMT*, and *KDM1B*, had the lowest *P*-values of 1.251x10^-23^, 4.639x10^-23^, and 7.68x10^-11^ respectively; all these genes are involved in metabolism-related pathways [36,40,41]. Although containing no genome-wide significant SNPs, 3q25.33 appears to be an important genomic region for Horvath-EAA, with four significantly associated genes including *TRIM59* and *KPNA4*, which play roles in the immune system [42,43]. Two further significant genes are *FAIM* and *TERT*, whose functions include apoptosis and autophagy [33], and telomere length-associated ageing and apoptosis [44,45] respectively. Twelve genes were significantly associated with Hannum-EAA (**Table 2**, **S14 Table**). Genes of interest include *MTRNR2L7*, a neuroprotective and anti-apoptotic factor [46,47], and *TRIM46* and *MUC1*, both located at 1q22, and which are involved with innate immune system pathways [42,48]. The 4q24 cytogenetic band houses several genes significantly associated with Hannum-EAA: *MANBA* and *UBE2D3* have metabolic and innate immune system functions [32,49] while *CISD2* regulates autophagy and is involved in life span control [50,51]. Comparing the results of the gene-based association analyses of Horvath-based and Hannum-based EAA, there was no overlap in significantly associated genes. Manhattan plots and QQ plots for the gene-based analysis of both epigenetic age acceleration measures are shown in **S3 Fig and S4 Fig**.

**Table 2:** Results of MAGMA gene-based association analysis for Horvath-based and Hannum-based epigenetic age acceleration.

Phenotype	Gene	Chr	N_SNPs	*P*-value	Function/related pathways
Horvath-EAA	*SELP*	1	148	1.6883E-07	Immunoglobulin E responsiveness
	*EDARADD*	1	516	4.2627E-07	Innate immune system, cytokine signalling in immune system
	*GATA2*	3	72	1.0663E-07	Stem cell maintenance and hematopoietic development
	*ESYT3*	3	93	6.5973E-07	Metabolism, lipid transport
	*CEP70*	3	164	1.0891E-06	Organelle biogenesis and maintenance
	*FAIM*	3	49	2.4177E-08	Apoptosis and autophagy; regulates B-cell signalling and differentiation
	*PIK3CB*	3	175	2.52E-08	Coordinates cell functions e.g. proliferation, survival, migration
	*IFT80*	3	150	1.2622E-07	Organelle biogenesis and maintenance; intraflagellar transport
	*SMC4*	3	77	9.8909E-07	Changes in chromosome structure during mitotic segregation
	*TRIM59*	3	105	1.7737E-07	Multifunctional regulator for innate immune signalling pathways
	*KPNA4*	3	104	2.9437E-07	Cytokine signalling in immune system; protein transporter activity
	*TERT*	5	90	4.0455E-08	Roles in ageing and apoptosis; regulation of telomerase.
	*NHLRC1*	6	76	1.2512E-23	Clearance of toxic polyglucosan and protein aggregates; metabolism pathways
	*TPMT*	6	151	4.6385E-23	Drug metabolism—cytochrome P450; thiopurine S methyltransferase activity
	*KDM1B*	6	248	7.6758E-11	Metabolism of proteins, regulates histone lysing methylation
	*SCGN*	6	202	3.8379E-10	Calcium binding protein; neuroscience, Ca, cAMP and lipid signalling pathways
	*TMEM72*	10	123	1.1154E-06	Transmembrane protein
	*RFX4*	12	299	9.4786E-07	Transcriptional regulatory network in embryonic stem cell
	*RIC8B*	12	155	1.0988E-06	Can activate some G-alpha proteins; odorant signal transduction.
	*C12orf23/TMEM263*	12	98	6.321E-09	Transmembrane protein
	*ZNF70*	22	79	2.7934E-06	Transcriptional regulation; gene expression pathways
Hannum-EAA	*TRIM46*	1	27	2.66E-06	Innate immune system; cytokine signalling in immune system
	*MUC1*	1	15	7.33E-07	Cytokine signalling in immune system; bacterial infections in CF airways
	*MANBA*	4	190	1.31E-06	Glycosaminoglycan metabolism; innate immune system
	*UBE2D3*	4	154	1.19E-06	Metabolism of proteins; innate immune system
	*CISD2*	4	54	1.18E-06	Regulator of autophagy; life span control; glucose/energy metabolism pathways
	*SLC9B1*	4	217	2.56E-06	Sperm motility and fertility, ion channel transport
	*MTRNR2L7*	10	52	5.20E-07	Neuroprotective and antiapoptotic factor
	*ZNF248*	10	204	2.22E-07	Transcriptional regulation; gene expression pathways
	*ZNF25*	10	99	1.23E-08	Transcriptional regulation; gene expression pathways
	*ZNF33A*	10	159	8.29E-09	Transcriptional regulation; gene expression pathways
	*ZNF37A*	10	146	3.79E-10	Transcriptional regulation; gene expression pathways
	*DNTT*	10	131	2.51E-07	DNA double-strand break repair; hematopoietic cell lineage

Genome-wide significant results after Bonferroni correction for multiple testing (*P*<2.809x10^-6^) are reported. N_SNPs is the number of SNPs in the gene.

### Gene-set and pathway analysis

Using a competitive test of enrichment implemented in MAGMA v1.6, we did not identify any gene sets that were significantly associated with either Horvath-EAA or Hannum-EAA after Bonferroni correction for multiple testing. **S15 Table and S16 Table** show the top 100 gene-sets for Horvath-EAA and Hannum-EAA respectively.

### Genetic correlations

Several large-scale cohort studies have previously reported phenotypic associations between epigenetic age acceleration and a number of traits or health outcomes. To investigate whether these observed associations may be partly due to shared genetic variants influencing the traits, we conducted cross-trait LD score regression analysis of summary-level data [52], implemented in the online software LD Hub [53], to determine genetic correlations between Horvath-EAA/Hannum-EAA and a number of health and behavioural variables. The SNP-based genetic correlation between Horvath-EAA and Hannum-EAA was 0.571 (SE = 0.132, *P* = 1.605x10^-5^), suggesting a moderate overlap in the genetic factors influencing these two measures of epigenetic age acceleration. Of the 218 other health and behavioural traits investigated, none had a statistically significant genetic correlation (*P*_*FDR*_<0.05) with either Horvath-EAA or Hannum-EAA after applying false discovery rate correction (most significant correlation with Horvath-EAA: father's age at death, *P*_*FDR*_ = 0.160; with Hannum-EAA: waist-to-hip ratio, *P*_*FDR*_ = 0.065). This correction, however, may be overly conservative, as not all the tested traits are independent, with several being highly correlated. Nominally significant correlations (*P*_*uncorrected*_<0.05) were found with a number of traits (**Table 3**).

**Table 3:** Nominally significant genetic correlations (*P*_*uncorrected*_<0.05) between Horvath-EAA/Hannum-EAA and other health and behavioural traits.

Phenotype	Trait	Genetic Correlation	SE	*P*-value
Horvath-EAA	Fathers age at death	-0.472	0.144	0.001
	Urate	0.278	0.089	0.002
	Waist-to-hip ratio	0.194	0.064	0.002
	Waist circumference	0.178	0.064	0.005
	ICV	-0.403	0.163	0.013
	Forced expiratory volume in 1 second (FEV1)/Forced Vital capacity(FVC)	-0.170	0.078	0.030
	Extreme waist-to-hip ratio	0.306	0.146	0.036
	Child birth weight	-0.274	0.131	0.037
	Childhood IQ	-0.317	0.152	0.037
	Leucine	0.450	0.222	0.043
	Glycoprotein acetyls; mainly a1-acid glycoprotein	0.360	0.183	0.049
Hannum-EAA	Waist-to-hip ratio	0.225	0.062	0.0003
	Waist circumference	0.210	0.067	0.002
	Parents age at death	-0.455	0.148	0.002
	Type 2 Diabetes	0.331	0.114	0.004
	Years of schooling 2013	-0.231	0.083	0.005
	Years of schooling 2016	-0.162	0.058	0.006
	Birth weight	0.211	0.079	0.007
	HDL cholesterol	-0.210	0.082	0.010
	Former vs Current smoker	-0.330	0.130	0.011
	Forced expiratory volume in 1 second (FEV1)	-0.239	0.095	0.012
	Forced expiratory volume in 1 second (FEV1)	-0.371	0.149	0.013
	Hip circumference	0.163	0.066	0.013
	Intelligence	-0.169	0.070	0.016
	Cigarettes smoked per day	0.326	0.138	0.018
	Ever vs never smoked	0.202	0.088	0.022
	College completion	-0.195	0.086	0.023
	Age of first birth	-0.156	0.070	0.026
	HOMA-B	0.305	0.137	0.026
	Fasting insulin main effect	0.237	0.107	0.027
	HbA1C	-0.277	0.126	0.028
	Childhood IQ	-0.286	0.130	0.028
	Fathers age at death	-0.287	0.131	0.028
	Triglycerides	0.136	0.065	0.036
	Phospholipids in medium LDL	-0.410	0.200	0.040
	Free cholesterol in large LDL	-0.485	0.237	0.041
	Anorexia Nervosa	-0.152	0.074	0.041
	Amyotrophic lateral sclerosis	0.363	0.178	0.042
	Obesity class 1	0.135	0.067	0.042
	Years of schooling (proxy cognitive performance)	-0.171	0.084	0.042
	Phospholipids in large LDL	-0.471	0.232	0.043
	Phospholipids in very small VLDL	-0.373	0.184	0.043
	Free cholesterol in IDL	-0.439	0.220	0.046
	Celiac disease	-0.265	0.134	0.047
	Extreme waist-to-hip ratio	0.285	0.145	0.048
	Total cholesterol in large LDL	-0.430	0.219	0.049

Genetic correlations were determined using bivariate Linkage Disequilibrium score regression implemented in the online software LD Hub. SE is the standard error of the genetic correlation estimate; *P*-value is the association *P-*value for the genetic correlation estimate; ICV–intracranial volume; LDL–low density lipoprotein; IDL–intermediate density lipoprotein; VLDL–very low density lipoprotein.

Both epigenetic age acceleration measures had nominally significant positive genetic correlations with a range of traits pertaining to adiposity, and negative correlations with father’s age at death and childhood IQ. Nominally significant genetic correlations were observed between Hannum-EAA, but not Horvath-EAA, and a wide range of traits including measures relating to education, smoking behaviour, various lipid- and cholesterol-related measures, diabetes and related glycemic measures, and parent’s age at death. Some of these results have previously been reported [19,20], but many are novel. The current study did, however, fail to replicate a number of previously reported correlations, including with age at menopause [20]. Details of the genetic correlations of all the tested traits with Horvath-EAA and Hannum-EAA are given in **S17 Table and S18 Table**, respectively.

## Discussion

This study investigated genetic markers of epigenetic ageing in a sample of 13,493 individuals of European ancestry. We examined genetic determinants of both Horvath-based (adjusted for the composition of age-related blood cells) and Hannum-based (immune system-associated) epigenetic age acceleration, sometimes referred to as ‘intrinsic’ and ‘extrinsic’ epigenetic age acceleration, to gain insight into the regulation of epigenetic ageing. We report several novel findings in addition to replicating a sub-set of previous results. The meta-analysis of Horvath-EAA identified ten independent associated SNPs, doubling the number reported to date, and highlighted 21 genes involved in Horvath-based epigenetic ageing. A single genome-wide significant variant was identified for Hannum-EAA, along with 12 implicated genes. We uncovered limited evidence of functionality within some associated genomic loci, with many SNPs located in regions of open chromatin and a smaller number in regulatory regions. Some loci also contained regions where genetic variation is predicted to be deleterious.

It has been hypothesised that in some cases DNA methylation could be a candidate mechanism for mediating genetic effects on ageing-related phenotypes [54]. Intriguingly, four of the ten Horvath-EAA-associated SNPs are mQTL for CpGs used in the Horvath/Hannum epigenetic clocks. A possible interpretation of this is that the functional mechanism by which these SNPs influence the rate of biological ageing is via altering methylation levels.

A number of the genes significantly associated with Horvath-EAA are related to metabolism (*NHLRC1*, *TPMT*, *KDM1B*, and *ESYT3*), consistent with several studies reporting phenotypic associations between Horvath-based EAA and metabolic syndrome characteristics and supporting the suggestion of a role in tracking metabolic ageing [15,19]. Others are involved in immune system pathways (*TRIM59*, *KPNA4*, *EDARADD*), while several have roles in cellular processes linked to ageing: apoptosis and autophagy (*FAIM*), ageing and autophagy (*TERT*), and coordinating vital cell functions (*PIK3CB*). *PIK3CB* plays a role in the signal transduction of insulin and insulin-like pathways [55], and genetic variants at this locus have been related to insulin-like growth factor levels in plasma, and human longevity [56].

Genes associated with Hannum-based EAA, often referred to as immune system ageing, include several involved in innate immune system pathways (e.g. *TRIM46* and *MUC1*) or with metabolic and immune system functions (*MANBA*, *UBE2D3*). Other associated genes of interest include those with roles relating to ageing and longevity: *MTRNR2L7* is a neuroprotective and anti-apoptotic factor, and *CISD2* regulates autophagy and is a fundamentally important regulator of lifespan. Mouse studies indicate that *CISD2* ameliorates age-associated degeneration of skin, skeletal muscle, and neurons, protects mitochondria from age-related damage and functional decline, and attenuates age-associated reduction in energy metabolism [57], while *CISD2* deficiency leads to a number of phenotypic features suggestive of premature ageing [58].

Our LD score regression analysis replicated the positive genetic correlations with central adiposity reported by Lu et al. (2018) at nominal significance levels, supporting the suggestion that observed phenotypic associations [15,19] may result in part from a shared genetic aetiology. We did not, however, replicate previously reported correlations between Horvath-EAA and metabolic disease-related traits or diabetes, and found these traits to be correlated with Hannum-EAA at only nominal significance levels in our larger sample [20]. We also found no correlation between epigenetic age acceleration and age at menopause. Nominally significant genetic correlations between Hannum-based, but not Horvath-based, epigenetic age acceleration, and lifestyle factors such as smoking behaviour and education level, provide some evidence for a genetic basis underlying the phenotypic results we reported previously [19], and provide tentative support for the hypothesis that Hannum-based epigenetic ageing is relatively sensitive to changes in environment and lifestyle. Father’s age at death, a rough proxy for lifespan [59], was nominally significantly correlated with both EAA measures, and parents’ age at death was additionally correlated with Hannum-EAA, consistent with a body of work demonstrating robustly that EAA predicts life span [10,12]. Aside from these, genetic correlations with age-related traits were surprisingly few: it is possible that this could reflect an overly conservative correction for the multiple tests carried out, or low statistical power, rather than a genuine lack of correlations (**S4 Table**). While the mean *χ*^2^ values (1.059 and 1.054 for Horvath-EAA and Hannum-EAA respectively) indicate a sufficient level of polygenicity within the dataset for use with LD score regression, the heritability Z-scores for Horvath-EAA and Hannum-EAA are 3.69 and 4.91 respectively. The recommendation is that genetic correlation analysis should be restricted to GWAS with a heritability Z-score of 4 or more, on the grounds of interpretability and power [53], so the Horvath-based results particularly should be interpreted with caution.

This study of epigenetic age acceleration benefits from having a large sample size. Increasing GWAS sample size increases the power to detect associated loci, and is often achieved, as in this case, by combining smaller studies in a meta-analysis. Meta-analytic GWAS are, however, sometimes hampered by differences in how a trait is measured between individual studies. In this instance, use of the online calculator to calculate the EAA measures and using the same algorithm and output columns for each study, mitigates this. The current study comprises only individuals of European ancestry, which confers a further advantage as epigenetic ageing rates have been shown to differ between ethnicities [60].

Despite the large sample overlap, some results of this study differ from those reported by Lu et al. (2018). One reason for this could be that only European-ancestry individuals were included in this analysis whereas the Lu study reports results from a mixed ancestry sample. Another likely contributing factor is the age ranges involved: the GS cohort, not included in Lu’s analysis but which makes up 38% of the total sample in the current study, has a mean age of 48.5 years, 14.4 years younger than the mean age of the remaining cohorts. Given that epigenetic age changes over the life course, although not necessarily in parallel with chronological age, this could help explain the discrepancies between the studies.

There are a number of limitations which should be considered when interpreting the results of this study. This is the largest meta-analysis of genetic determinants of epigenetic age acceleration to date, however, while large for these phenotypes, the size of the sample studies here is still small in terms of genome-wide analysis of polygenic traits. As only European-ancestry individuals were included, the results are not generalisable to other ethnicities. The MAGMA gene-based analysis identified a number of biologically plausible associated genes for both EAA measures; however, while many of these genes are located in the same genomic regions as the significantly associated SNPs, this should not be taken as evidence that the SNP association is effected through the gene. Identifying effector transcripts for GWAS variants is a difficult and as yet unresolved problem, and our knowledge of how these genes may affect the activity of the SNPs is limited. In addition, MAGMA does not take into account information from methylation QTL to help identify relevant genes; future work should place more emphasis on the role of mQTL. The lack of significant genetic correlations between EAA and age-related traits may reflect low statistical power (the heritability Z-score of 3.69 for Horvath-EAA falls below the recommended lower threshold of 4 for genetic correlation analysis) or overly stringent correction for multiple comparisons (FDR correction was applied over the 218 tested traits, however not all of these were independent) rather than a true absence of shared genetic aetiology. Finally, while we have identified a number of SNPs and genes significantly associated with EAA, including genes already known to be related to ageing, the analyses presented here fall short of providing a mechanistic explanation for how these variants and genes act to influence biological age. This study should be considered as 'discovery' research, with a comprehensive investigation of the functional and biological mechanisms behind the SNP and gene associations being a direction for future work.

Horvath-based and Hannum-based epigenetic age acceleration are thought to represent different aspects of ageing. Hannum-EAA has been described as a biomarker of immune system ageing, and has been found to be associated with a wide range of traits [15,19], indicating a sensitivity to variations in environment and lifestyle. By contrast, Horvath-EAA is considered to be a fundamental, intrinsic cellular ageing process, largely unrelated to lifestyle factors, although associations with a range of metabolic syndrome characteristics suggest a role in tracking metabolic ageing processes. Our results reflect this to a large degree, with more nominally significant genetic correlations found with Hannum-EAA than Horvath-EAA, including items relating to education, smoking, intelligence, and various cholesterol measures. Meanwhile the greater number of significant variants, genomic loci, and genes associated with Horvath-EAA are consistent with the hypothesis that this measure of 'cell-intrinsic' ageing is less related to lifestyle and more under genetic control, and thus more likely to remain relatively stable. Despite these differences, however, our results indicate some common features. The significant genetic correlation of 0.57 between the two measures suggests a moderate overlap in the genetic factors influencing the two phenotypes despite the biomarkers being based on almost entirely distinct CpG sets. Both also appear to be influenced by genes associated with metabolic and immune system pathways, although the specific genes involved are different.

### Conclusions

This study provided insight into the genetic determinants of differential biological ageing through the identification of genes and genetic variants associated with epigenetic age acceleration. We doubled the number of SNPs associated with Horvath-EAA reported to date, and report 21 genes significantly associated with this phenotype, including *PIK3CB*, linked to human longevity. We identified 12 Hannum-EAA-associated genes, one of which, *CISD2*, has a fundamental role in lifespan control. Our results also highlighted differences in the genetic architecture of the Horvath-based and Hannum-based EAA measures, with no genome-wide significant SNPs or genes common to the two, providing substantial support for the hypothesis that they represent different aspects of ageing.

While the genetic information coded by our DNA sequence remains largely fixed throughout the lifetime, the expression of our genes is primarily regulated by epigenetic factors, which change over time. Epigenetic age increases with, but not in parallel with, chronological age; individual differences in the rate of epigenetic ageing potentially explain why trajectories of ageing differ between individuals. Understanding what causes these differences could potentially inform therapeutic interventions to delay the onset of age-related decline and improve ageing outcomes.

## Methods

### Ethics statement

Generation Scotland received ethical approval from the NHS Tayside Committee on Medical Research Ethics (REC Reference Number: 05/S1401/89). GS has also been granted Research Tissue Bank status by the Tayside Committee on Medical Research Ethics (REC Reference Number: 10/S1402/20), providing generic ethical approval for a wide range of uses within medical research. All participants provided written informed consent. Details of ethics approval and consent to participate for the cohorts included in the Lu et al. (2018) study can be found in their publication.

### Generation Scotland cohort

We carried out genome-wide association analyses of Horvath-EAA and Hannum-EAA in a subset of individuals (n = 5,100) from the Generation Scotland: Scottish Family Health Study (GS) for whom both genetic and DNA methylation data were available. GS is a family- and population-based cohort recruited via general medical practices across Scotland; the recruitment protocol and sample characteristics are described in detail elsewhere [61,62]. In brief, the full cohort comprises 23,960 individuals aged between 18 and 98 years. Pedigree information was available for all participants, detailed socio-demographic and clinical data were collected, and biological samples were taken for genotyping.

### DNA methylation and derivation of epigenetic age acceleration variables in GS

DNA methylation data were obtained from peripheral blood (n = 5,091) or saliva (n = 10) samples for 5,101 individuals from GS, with quality control checks carried out using standard methods outlined in **S1 Text**, and described in full elsewhere [19]. After quality control (QC), the dataset comprised beta-values for 860,928 methylation loci. Methylation-based age estimates (DNAm age) and epigenetic age acceleration variables (Horvath-EAA and Hannum-EAA, described in **S1 Text**) were obtained from the online DNA Methylation Age Calculator (https://dnamage.genetics.ucla.edu/) developed by Horvath [8]. Normalised DNA methylation beta-values were submitted to the calculator, using the 'Advanced Analysis for Blood Data' option, and undergoing further normalisation within the calculator algorithm to make the data comparable to the training data of the epigenetic clock. One individual was flagged by the calculator as having a gender mismatch, and was therefore omitted from downstream analysis, leaving a total of 5,100 individuals for the GWAS of Horvath-EAA and Hannum-EAA in GS. Blood cell abundance measures were also estimated by the online calculator, based on DNA methylation levels, as described previously [63].

### Genotyping, imputation, and quality control in GS

An overview of biological sample collection, DNA extraction, genotyping, imputation using the Haplotype Research Consortium reference panel (v1.1), and quality control for GS is included in **S1 Text**; full details have been described previously [64]. A total of 20,032 individuals passed all quality control thresholds. Following the removal of monomorphic or multiallelic variants and SNPs with a low imputation quality or a minor allele frequency below 1%, an imputed dataset with 8,633,288 hard called variants remained to be used in the genome-wide association analysis.

### GWAS of Horvath-EAA and Hannum-EAA in GS

GWAS of Horvath-EAA and Hannum-EAA in GS were conducted using mixed linear model based association (MLMA) analysis [65], implemented in GCTA (Genome-wide Complex Trait Analysis) (v1.25) [66], and adjusting for sex to account for the higher epigenetic age acceleration in men than in women [7,12,60]. In order to account for population stratification, it is common to conduct ancestry-informative principal components analysis on the population in question, and use a number of the top-ranking principal components (PCs) from this analysis as covariates in the GWAS. However, as GS is a family-based sample, we employed a different approach to capture population structure. In place of PCs, two genomic relationship matrices (GRMs) were included in the GWAS, as this method has been shown to account for potential upward biases due to excessive relationships, and thus allows the inclusion of closely and distantly related individuals in genetic analyses [67]. The first GRM included pairwise relationship coefficients for all individuals, while the second had off-diagonal elements <0.05 set to 0; full details of the methods involved and construction of the GRMs is given elsewhere [68]. The results of univariate LD score regression analysis [26] (**S4 Table**) indicate that the two GRMs adequately accounted for population stratification, so it was not necessary to include ancestry-informative PCs in the GWAS.

### GWAS meta-analysis of Horvath-EAA and Hannum-EAA

We obtained summary statistics from the largest European-ancestry analysis of epigenetic age acceleration to date (n = 8,393, Lu et al., 2018, summary information in **S19 Table**), and meta-analysed these with GS (details above). We chose not to include available data from non-European samples, despite the advantages of increased sample size, as different ethnicities have been shown to have different epigenetic ageing rates [60]. Association summary statistics from the GWAS of the two EAA phenotypes in GS and the Lu et al. study were meta-analysed using the inverse variance-weighted approach, which weights effect sizes by sampling distribution. This analysis was implemented in METAL [69], conditional on each variant being available in both samples. As SNPs which co-located with CpGs from the Hannum- or Horvath-based DNAm age predictors had already been excluded from Lu et al.'s analysis, it was not necessary to repeat this step. This resulted in 5,932,107 genetic variants for Horvath-EAA and 5,931,171 variants for Hannum-EAA, in a meta-analysis dataset containing 13,493 participants.

The meta-analytic summary statistics produced by METAL were uploaded to FUMA (fuma.ctglab.nl) [27], which identified index SNPs and genomic risk loci related to epigenetic age acceleration. FUMA selects independent significant SNPs based on their having a genome-wide significant *P*-value (*P*<5x10^-8^) and being independent from each other (*r*^2^<0.6 by default) within a 250kb window. The European subset of the 1000 Genomes phase 3 reference panel [70] was used to map LD. SNPs in LD with these independent significant SNPs (*r*^2^≥0.6) within a 250kb window, and which have a minor allele frequency (MAF)>1% within the 1000 Genomes reference panel, were included for further annotation and used for gene prioritization. A subset of the independent significant SNPs, those in LD with each other at *r*^2^<0.1 within a 250kb window, were identified as lead SNPs. Genomic risk loci, including all independent signals that were physically close or overlapping in a single locus, were identified by merging any lead SNPs that were closer than 250kb apart (meaning that a genomic risk locus could contain multiple lead SNPs, with each locus represented by the lead SNP with the lowest *P*-value in that locus).

Conditional analysis was implemented using GCTA software [66] to ascertain whether associated genetic loci harboured more than one independent causal variant, conditioning on the lead SNP at the locus and using GS as the reference panel for inferring the LD pattern. SNPs which remained significantly associated (*P*<5x10^-8^) with the phenotype after conditioning on the lead SNP were considered to be further independent associated variants.

Manhattan plots and quantile-quantile plots were generated in R version 3.2.3 using the 'qqman' package, and regional SNP association results were visualised with LocusZoom [21]. SNPs which surpassed the threshold for genome-wide significance in our meta-analyses were checked against the NHGRI-EBI catalog of published GWAS [71,72] (www.ebi.ac.uk/gwas/) to determine whether they had previously been observed in association analysis.

### Methylation quantitative trait loci

To ascertain whether the genome-wide significant associations from the Horvath-EAA and Hannum-EAA GWAS are confounded by methylation quantitative trait loci, we checked for SNP-CpG pairings in the mQTL database, a catalogue of the genetic influences on DNA methylation (mQTLdb, [25]). The independent significant SNPs from both GWAS were input to the database, using the MatrixEQTL database setting, which contains all associations below 1x10^-7^, and assessing all five time points (birth, adolescence, childhood, middle age, and pregnancy). A distance greater than or equal to 1 Mb was considered to be *trans*.

### Heritability analysis

To estimate the SNP-based heritability for Horvath-EAA and Hannum-EAA, univariate Linkage Disequilibrium score regression [26] was applied to the GWAS summary statistics for both measures. This method also provides metrics to evaluate the proportion of inflation in the test statistics caused by confounding biases such as residual population stratification, relative to genuine polygenicity. We used pre-computed LD scores, estimated from the European-ancestry samples in the 1000 Genomes Project [73].

### SNP functional annotation

Functional annotation, using all SNPs located within the genomic risk loci which were nominally significant (*P*<0.05), had a MAF≥1%, and were in LD of *r*^2^≥0.6, was carried out in FUMA v1.3.0 [27]. In order to investigate the functional consequences of variation at these SNPs, they were first matched (based on chromosome, base pair position, reference and non-reference alleles) to a database containing functional annotations from a number of repositories:

- ANNOVAR (Annotate Variation) categories [74], used to identify a SNP's function and determine its position within the genome.
- Combined Annotation Dependent Depletion (CADD) scores [28], a measure of the deleteriousness of genetic variation at a SNP to protein structure and function, with higher scores indicating more deleterious variants.
- RegulomeDB (RDB) scores [29], based on data from eQTL as well as chromatin marks, with lower scores given to variants with the greatest evidence for having regulatory function.
- Chromatin states [75–77], indicating the level of accessibility of genomic regions, described on a 15 point scale, where lower chromatin scores indicate a greater level of accessibility to the genome at that site; generally, between 1 and 7 is considered an open chromatin state.

### Gene-based analysis

Gene-based analysis was performed for each phenotype using the results of our association analysis, using default settings in MAGMA v1.6 [39], integrated within the FUMA web application. Summary statistics of SNPs located within protein-coding genes were aggregated to assess the simultaneous effect of all SNPs in the gene on the phenotype. The European panel of the 1000 Genomes phase 3 data was used as a reference panel to account for LD [70]. Genetic variants were assigned to protein-coding genes obtained from Ensembl build 85, resulting in 17,798 genes being analysed. After Bonferroni correction (α = 0.05/17,798), a threshold for genome-wide significant genes was defined at *P*<2.809×10^−6^.

### eQTL and colocalisation analysis

The independent genome-wide significant SNPs identified in the meta-analyses of Horvath-EAA and Hannum-EAA were assessed to determine whether they were potential eQTL, by mapping SNPs to genes if allelic variation at the SNP is associated with expression levels of the gene. This analysis was carried out using data from the Genotype Tissue Expression portal (GTEx) v7 [30], integrated within the FUMA web application. GTEx uses gene expression data from 48 different types of human tissue, linked to genotype data to provide information on eQTL. Since Horvath-EAA is derived from the pan-tissue Horvath epigenetic clock, eQTL analysis of the ten Horvath-EAA-associated SNPs used all the available tissue types in GTEx. Analysis for the Hannum-EAA SNP, however, was restricted to only the blood tissue types, as the Hannum epigenetic clock is specific to blood samples. eQTL mapping carried out within FUMA maps SNPs to genes which likely affect expression of those genes within 1Mb, i.e. *cis*-eQTL. Although FUMA contains all SNP-gene pairs of *cis*-eQTL, including non-significant associations, we limited our analysis to significant SNP-gene pairs, with a false discovery rate (FDR) ≤ 0.05 used as the cut-off to define significant eQTL associations.

To further investigate the potential regulatory functions of the identified SNPs, we carried out colocalisation analysis to determine whether the SNPs are mediated through gene expression. We integrated our GWAS results with *cis*-eQTL data from the eQTLGen Consortium (https://www.eqtlgen.org/) [38], using a Bayesian method, 'coloc' [37], which evaluates whether the GWAS and eQTL associations best fit a model in which the same SNP is associated with both EAA and *cis* gene expression. This method, implemented in the 'coloc' package in R, tests pairwise colocalisation of SNPs in significant genomic regions in the GWAS with eQTLs, and generates posterior probabilities for each locus by weighing the evidence for competing hypotheses of no causal variants for either trait, causal variants for one trait only, independent causal variants influencing the two traits, or a shared causal SNP. We extracted summary statistics from the Horvath-EAA/Hannum-EAA meta-analytic GWAS results for all SNPs in a +/- 200 kb region around each genome-wide significant SNP, and extracted equivalent summary data for the same region in the eQTL analysis. Using the default prior probabilities in ‘coloc’, pairwise colocalisation was then tested between each GWAS-eQTL pair, with a posterior probability of ≥0.95 considered to be strong evidence in favour of a given hypothesis.

### Gene-set analysis

To assess whether the Horvath-EAA and Hannum-EAA GWAS meta-analysis results are enriched for various gene-sets and provide insight into the involvement of specific biological pathways in the genetic aetiology of the phenotype, the gene-based analysis results were used to perform competitive gene-set and pathway analysis using default parameters in MAGMA v1.6, integrated within FUMA. The reference genome was 1000 genomes phase 3. This analysis used gene annotation files from the Molecular Signatures Database v5.2 for "Curated gene sets", covering chemical and genetic perturbations, and Canonical pathways, and "GO terms", covering three ontologies: biological process, cellular components, and molecular function. A total of 10,894 gene-sets were examined for enrichment in Horvath-EAA and Hannum-EAA, with a Bonferroni correction applied to control for multiple testing. Thus genome-wide significance was defined at *P =* 0.05/10,894 = 4.59x10^-6^.

### Genetic correlations

Cross trait LD score regression [52] was used to calculate genetic correlations between Horvath-based and Hannum-based EAA in our meta-analysis, and then between Horvath-EAA/Hannum-EAA and 218 other behavioural and disease-related traits for which GWAS summary data were available through LD Hub [53]; traits derived from non-Caucasian or mixed ethnicity samples were removed prior to analysis. This method exploits the correlational structure of SNPs across the genome and uses test statistics provided from GWAS summary estimates to calculate the genetic correlations between traits [52]. We checked whether our meta-analysis datasets had sufficient evidence of a polygenic signal, indicated by a heritability Z-sc*o*re of >4 and a mean χ2 statistic of >1.02 [52]. By default, a MAF filter of >1% was applied, and indels and strand ambiguous SNPs were removed. We filtered to HapMap3 SNPs, and SNPs whose alleles did not match those in the 1000 Genomes European reference sample were removed. LD scores and weights for use with European populations were downloaded from (https://github.com/bulik/ldsc). We did not constrain the intercepts in our analysis, as we could not quantify the exact amount of sample overlap between cohorts. False discovery rate correction was applied across the 218 traits to correct for multiple testing [78].

## Supporting information

S1 Text Supplementary information. S1 Text contains further information on the Hannum and Horvath epigenetic clocks, measures of epigenetic age and epigenetic age acceleration, DNA methylation in GS, derivation of epigenetic age and epigenetic age acceleration variables in GS, genotyping, imputation, and quality control in GS.
(DOCX)

S1 Table Summary of age and estimated epigenetic age variables in Generation Scotland. (XLSX)

S2 Table Independent variants with a genome-wide significant association (*P*<5x10^-8^) with epigenetic age acceleration in the Generation Scotland cohort. (XLSX)

S3 Table Independent variants with a *P*-value <5x10^-8^ for association with Horvath-EAA/Hannum-EAA in the Lu et al. sample, and their corresponding effect size and significance in the Generation Scotland cohort. (XLSX)

S4 Table Estimated polygenicity and SNP-based heritability using LD score regression. (XLSX)

S5 Table Full details of independent variants with a genome-wide significant association (*P*<5x10^-8^) with Horvath-based or Hannum-based epigenetic age acceleration, ordered by chromosomal location. (XLSX)

S6 Table Independent variants with a *P*-value <5x10^-8^ for association with Horvath-EAA/Hannum-EAA in the meta-analysis, and their corresponding effect size and significance in the Generation Scotland and Lu samples. (XLSX)

S7 Table Summary of the independent variants significantly associated with either Horvath-EAA or Hannum-EAA, and their association with both epigenetic age acceleration measures. (XLSX)

S8 Table Horvath-EAA-associated SNPs which act as methylation quantitative trait loci for CpGs included in the Horvath or Hannum epigenetic clocks. (XLSX)

S9 Table Summary of Horvath-EAA and Hannum-EAA associated SNPs which act as methylation QTL for CpGs in the mQTL database. (XLSX)

S10 Table Functional annotation of all SNPs in LD (r^2^≤0.6) with FUMA-identified independent significant SNPs for Horvath-EAA. (XLSX)

S11 Table Functional annotation of all SNPs in LD (r^2^≤0.6) with FUMA-identified independent significant SNPs for Hannum-EAA. (XLSX)

S12 Table Expression quantitative trait loci identified by analysis of independent significant variants for Horvath-EAA and the significance of their expression in the specified tissues. (XLSX)

S13 Table Results of colocalisation analysis in the regions surrounding genome-wide significant SNPs for Horvath-EAA and Hannum-EAA. (XLSX)

S14 Table Genome-wide significant gene-based results (*P*<2.809x10^-6^) obtained by MAGMA gene-based association analyses of Horvath-EAA and Hannum-EAA. (XLSX)

S15 Table Most associated gene sets for the GWAS meta-analysis of Horvath-EAA. (XLSX)

S16 Table Most associated gene sets for the GWAS meta-analysis of Hannum-EAA. (XLSX)

S17 Table Genetic correlations between Horvath-EAA and 218 other health and behavioural traits, using bivariate Linkage Disequilibrium score regression. (XLSX)

S18 Table Genetic correlations between Hannum-EAA and 218 other health and behavioural traits, using bivariate Linkage Disequilibrium score regression. (XLSX)

S19 Table Overview of study datasets. (XLSX)

S1 Fig QQ plots for the GWAS of Horvath-EAA and Hannum-EAA in GS, showing the expected distribution of GWAS test statistics, -log10(p), versus the observed distribution. (DOCX)

S2 Fig Regional association plots for Horvath-EAA and Hannum-EAA associated SNPs. Regional association plots for nine independent significantly associated SNPs for Horvath-EAA (A-I) and the single independent significantly associated SNP for Hannum-EAA (J), showing LD with SNPs in the surrounding region. Plots were produced in LocusZoom. The SNP association P-value is given on the y-axis, and SNP position, with gene annotation, on the x-axis. LD calculations are taken from hg19/1000 Genomes European build. Individual SNPs are coloured according to the strength of LD (r2) with the lead SNP. The highest association signal in each panel, highlighted in violet, are as follows:
A: rs1011267, an intronic SNP in C1orf112 on chromosome 1; B: rs79070372, a non-coding transcript variant on chromosome 3 (closest genes GATA2/AS1); C: rs388649, an intronic SNP in PIK3CB on chromosome 3; D: rs6440667, an intronic SNP in LINC01214 on chromosome 3; E: rs2736099, an intronic SNP in TERT on chromosome 5; F: rs76244256, an intron variant in TPMT on chromosome 6 and the top ranking SNP for association with Horvath-EAA *This genomic locus contains a second independent associated SNP, intergenic variant rs7744541 (nearest gene NHLRC1), which remained significantly associated (P<5x10-8) with Horvath-EAA after conditioning on the lead SNP; G: rs4712953, an intronic SNP in SCGN on chromosome 6; H: rs10778517, a SNP of unknown function on chromosome 12 (nearest genes RP11-412D9.4 and TMEM263); I: rs62078811, an intron variant in STXBP4 on chromosome 17; J: rs1005277, the single independent Hannum-EAA significant associated SNP, a SNP of unknown function on chromosome 10 (nearest gene ZNF25).
(DOCX)

S3 Fig Manhattan plots for the MAGMA gene-based association analysis for the GWAS meta-analysis (n = 13,493) of Horvath-based epigenetic age acceleration and Hannum-based epigenetic age acceleration, with—log_10_ transformed *P*-values for each gene plotted against chromosomal location. The dotted line denotes genome-wide significance, defined at *P* = 0.05/17798 = 2.809x10^-6^. Genes whose *P*-value reached genome-wide significance are labelled on the plots.
(DOCX)

S4 Fig QQ plots for the gene-based association analyses of Horvath-EAA and Hannum-EAA, showing the expected distribution of test statistics, -log10(p), versus the observed distribution. (DOCX)

## References

[1] Niccoli T, Partridge L. Ageing as a Risk Factor for Disease. Curr Biol [Internet]. 2012 9 11 [cited 2018 Jun 4];22(17):R741–52. Available from: https://www.sciencedirect.com/science/article/pii/S0960982212008159?via%3Dihub 10.1016/j.cub.2012.07.024 22975005

[2] Rode L, Nordestgaard BG, Bojesen SE. Peripheral Blood Leukocyte Telomere Length and Mortality Among 64 637 Individuals From the General Population. JNCI J Natl Cancer Inst [Internet]. 2015 6 1 [cited 2018 Sep 25];107(6). Available from: https://academic.oup.com/jnci/article-lookup/doi/10.1093/jnci/djv074 10.1093/jnci/djv074 25862531

[3] Beck S, Rakyan VK. The methylome: approaches for global DNA methylation profiling. Trends Genet [Internet]. 2008 5 1 [cited 2018 May 14];24(5):231–7. Available from: https://www.sciencedirect.com/science/article/pii/S0168952508000577?via%3Dihub 10.1016/j.tig.2008.01.006 18325624

[4] Bollati V, Schwartz J, Wright R, Litonjua A, Tarantini L, Suh H, et al Decline in genomic DNA methylation through aging in a cohort of elderly subjects. Mech Ageing Dev [Internet]. 2009 4 [cited 2018 May 14];130(4):234–9. Available from: http://www.ncbi.nlm.nih.gov/pubmed/19150625 10.1016/j.mad.2008.12.003 19150625 PMC2956267

[5] Christensen BC, Houseman EA, Marsit CJ, Zheng S, Wrensch MR, Wiemels JL, et al Aging and Environmental Exposures Alter Tissue-Specific DNA Methylation Dependent upon CpG Island Context. Schübeler D, editor. PLoS Genet [Internet]. 2009 8 14 [cited 2018 May 14];5(8):e1000602 Available from: 10.1371/journal.pgen.1000602 19680444 PMC2718614

[6] Shah S, McRae AF, Marioni RE, Harris SE, Gibson J, Henders AK, et al Genetic and environmental exposures constrain epigenetic drift over the human life course. Genome Res [Internet]. 2014 11 1 [cited 2018 May 14];24(11):1725–33. Available from: http://www.ncbi.nlm.nih.gov/pubmed/25249537 10.1101/gr.176933.114 25249537 PMC4216914

[7] Hannum G, Guinney J, Zhao L, Zhang L, Hughes G, Sadda S, et al Genome-wide methylation profiles reveal quantitative views of human aging rates. Mol Cell [Internet]. 2013 1 24 [cited 2018 May 14];49(2):359–67. Available from: http://www.ncbi.nlm.nih.gov/pubmed/23177740 10.1016/j.molcel.2012.10.016 23177740 PMC3780611

[8] Horvath S. DNA methylation age of human tissues and cell types. Genome Biol [Internet]. 2013 12 10 [cited 2018 May 14];14(10):R115 Available from: http://genomebiology.biomedcentral.com/articles/10.1186/gb-2013-14-10-r115 10.1186/gb-2013-14-10-r115 24138928 PMC4015143

[9] Marioni RE, Shah S, McRae AF, Ritchie SJ, Muniz-Terrera G, Harris SE, et al The epigenetic clock is correlated with physical and cognitive fitness in the Lothian Birth Cohort 1936. Int J Epidemiol [Internet]. 2015 8 1 [cited 2018 May 14];44(4):1388–96. Available from: https://academic.oup.com/ije/article-lookup/doi/10.1093/ije/dyu277 25617346 10.1093/ije/dyu277 PMC4588858

[10] Chen BH, Marioni RE, Colicino E, Peters MJ, Ward-Caviness CK, Tsai P-C, et al DNA methylation-based measures of biological age: meta-analysis predicting time to death. Aging (Albany NY) [Internet]. 2016 9 28 [cited 2018 May 14];8(9):1844–65. Available from: http://www.ncbi.nlm.nih.gov/pubmed/27690265 27690265 10.18632/aging.101020 PMC5076441

[11] Marioni RE, Suderman M, Chen BH, Horvath S, Bandinelli S, Morris T, et al Tracking the Epigenetic Clock Across the Human Life Course: A Meta-analysis of Longitudinal Cohort Data. J Gerontol A Biol Sci Med Sci [Internet]. 2019 1 1 [cited 2019 Jan 21];74(1):57–61. Available from: http://www.ncbi.nlm.nih.gov/pubmed/29718110 10.1093/gerona/gly060 29718110 PMC6298183

[12] Marioni RE, Shah S, McRae AF, Chen BH, Colicino E, Harris SE, et al DNA methylation age of blood predicts all-cause mortality in later life. Genome Biol [Internet]. 2015 1 30 [cited 2018 May 14];16(1):25 Available from: http://genomebiology.com/2015/16/1/25 25633388 10.1186/s13059-015-0584-6 PMC4350614

[13] Fagnoni FF, Vescovini R, Passeri G, Bologna G, Pedrazzoni M, Lavagetto G, et al Shortage of circulating naive CD8(+) T cells provides new insights on immunodeficiency in aging. Blood [Internet]. 2000 5 1 [cited 2019 Jan 21];95(9):2860–8. Available from: http://www.ncbi.nlm.nih.gov/pubmed/10779432 10779432

[14] Miller RA. The aging immune system: primer and prospectus. Science [Internet]. 1996 7 5 [cited 2019 Jan 21];273(5271):70–4. Available from: http://www.ncbi.nlm.nih.gov/pubmed/8658199 10.1126/science.273.5271.70 8658199

[15] Quach A, Levine ME, Tanaka T, Lu AT, Chen BH, Ferrucci L, et al Epigenetic clock analysis of diet, exercise, education, and lifestyle factors. Aging (Albany NY) [Internet]. 2017 2 14 [cited 2018 May 14];9(2):419–46. Available from: http://www.ncbi.nlm.nih.gov/pubmed/28198702 28198702 10.18632/aging.101168 PMC5361673

[16] Levine ME, Lu AT, Bennett DA, Horvath S. Epigenetic age of the pre-frontal cortex is associated with neuritic plaques, amyloid load, and Alzheimer’s disease related cognitive functioning. Aging (Albany NY) [Internet]. 2015 12 [cited 2018 May 14];7(12):1198–211. Available from: http://www.ncbi.nlm.nih.gov/pubmed/26684672 26684672 10.18632/aging.100864 PMC4712342

[17] Levine ME, Lu AT, Chen BH, Hernandez DG, Singleton AB, Ferrucci L, et al Menopause accelerates biological aging. Proc Natl Acad Sci U S A [Internet]. 2016 8 16 [cited 2018 May 14];113(33):9327–32. Available from: http://www.ncbi.nlm.nih.gov/pubmed/27457926 10.1073/pnas.1604558113 27457926 PMC4995944

[18] Horvath S, Ritz BR. Increased epigenetic age and granulocyte counts in the blood of Parkinson’s disease patients. Aging (Albany NY) [Internet]. 2015 12 [cited 2018 May 14];7(12):1130–42. Available from: http://www.ncbi.nlm.nih.gov/pubmed/26655927 26655927 10.18632/aging.100859 PMC4712337

[19] McCartney DL, Stevenson AJ, Walker RM, Gibson J, Morris SW, Campbell A, et al DNA methylation age acceleration and risk factors for Alzheimer’s disease. bioRxiv [Internet]. 2018 3 8 [cited 2018 Jun 12];278945. Available from: https://www.biorxiv.org/content/early/2018/03/08/278945 10.1016/j.dadm.2018.05.006 PMC6111045 30167451

[20] Lu AT, Xue L, Salfati EL, Chen BH, Ferrucci L, Levy D, et al GWAS of epigenetic aging rates in blood reveals a critical role for TERT. Nat Commun [Internet]. 2018 12 26 [cited 2018 May 14];9(1):387 Available from: http://www.nature.com/articles/s41467-017-02697-5 10.1038/s41467-017-02697-5 29374233 PMC5786029

[21] Pruim RJ, Welch RP, Sanna S, Teslovich TM, Chines PS, Gliedt TP, et al LocusZoom: regional visualization of genome-wide association scan results. Bioinformatics [Internet]. 2010 9 15 [cited 2018 May 15];26(18):2336–7. Available from: https://academic.oup.com/bioinformatics/article-lookup/doi/10.1093/bioinformatics/btq419 20634204 10.1093/bioinformatics/btq419 PMC2935401

[22] Twine NA, Harkness L, Kassem M, Wilkins MR. Transcription factor ZNF25 is associated with osteoblast differentiation of human skeletal stem cells. BMC Genomics [Internet]. 2016 [cited 2018 Nov 5];17(1):872 Available from: http://www.ncbi.nlm.nih.gov/pubmed/27814695 10.1186/s12864-016-3214-0 27814695 PMC5097439

[23] Bell JT, Pai AA, Pickrell JK, Gaffney DJ, Pique-Regi R, Degner JF, et al DNA methylation patterns associate with genetic and gene expression variation in HapMap cell lines. Genome Biol [Internet]. 2011 1 20 [cited 2019 Jun 15];12(1):R10 Available from: http://genomebiology.biomedcentral.com/articles/10.1186/gb-2011-12-1-r10 21251332 10.1186/gb-2011-12-1-r10 PMC3091299

[24] Gibbs JR, van der Brug MP, Hernandez DG, Traynor BJ, Nalls MA, Lai S-L, et al Abundant Quantitative Trait Loci Exist for DNA Methylation and Gene Expression in Human Brain. Flint J, editor. PLoS Genet [Internet]. 2010 5 13 [cited 2019 Jun 15];6(5):e1000952 Available from: https://dx.plos.org/10.1371/journal.pgen.1000952 20485568 10.1371/journal.pgen.1000952 PMC2869317

[25] Gaunt TR, Shihab HA, Hemani G, Min JL, Woodward G, Lyttleton O, et al Systematic identification of genetic influences on methylation across the human life course. Genome Biol [Internet]. 2016 12 31 [cited 2019 Jun 15];17(1):61 Available from: http://genomebiology.biomedcentral.com/articles/10.1186/s13059-016-0926-z 27036880 10.1186/s13059-016-0926-z PMC4818469

[26] Bulik-Sullivan BK, Loh P-R, Finucane HK, Ripke S, Yang J, Patterson N, et al LD Score regression distinguishes confounding from polygenicity in genome-wide association studies. Nat Genet [Internet]. 2015 2 2 [cited 2018 May 14];47(3):291–5. Available from: http://www.nature.com/doifinder/10.1038/ng.3211 25642630 10.1038/ng.3211 PMC4495769

[27] Watanabe K, Taskesen E, van Bochoven A, Posthuma D. Functional mapping and annotation of genetic associations with FUMA. Nat Commun [Internet]. 2017 12 28 [cited 2018 May 14];8(1):1826 Available from: http://www.nature.com/articles/s41467-017-01261-5 10.1038/s41467-017-01261-5 29184056 PMC5705698

[28] Kircher M, Witten DM, Jain P, O’Roak BJ, Cooper GM, Shendure J. A general framework for estimating the relative pathogenicity of human genetic variants. Nat Genet [Internet]. 2014 3 2 [cited 2018 May 14];46(3):310–5. Available from: http://www.nature.com/articles/ng.2892 10.1038/ng.2892 24487276 PMC3992975

[29] Boyle AP, Hong EL, Hariharan M, Cheng Y, Schaub MA, Kasowski M, et al Annotation of functional variation in personal genomes using RegulomeDB. Genome Res [Internet]. 2012 9 [cited 2018 May 14];22(9):1790–7. Available from: http://www.ncbi.nlm.nih.gov/pubmed/22955989 10.1101/gr.137323.112 22955989 PMC3431494

[30] Aguet F, Brown AA, Castel SE, Davis JR, He Y, Jo B, et al Genetic effects on gene expression across human tissues. Nature [Internet]. 2017 10 11 [cited 2018 May 15];550(7675):204–13. Available from: http://www.nature.com/doifinder/10.1038/nature24277 29022597 10.1038/nature24277 PMC5776756

[31] Saheki Y, Bian X, Schauder CM, Sawaki Y, Surma MA, Klose C, et al Control of plasma membrane lipid homeostasis by the extended synaptotagmins. Nat Cell Biol [Internet]. 2016 5 11 [cited 2018 Jun 11];18(5):504–15. Available from: http://www.nature.com/articles/ncb3339 10.1038/ncb3339 27065097 PMC4848133

[32] Belinky F, Nativ N, Stelzer G, Zimmerman S, Iny Stein T, Safran M, et al PathCards: multi-source consolidation of human biological pathways. Database (Oxford) [Internet]. 2015 [cited 2018 Jun 12];2015 Available from: http://www.ncbi.nlm.nih.gov/pubmed/25725062 10.1093/database/bav006 PMC4343183 25725062

[33] Segura MF, Sole C, Pascual M, Moubarak RS, Jose Perez-Garcia M, Gozzelino R, et al The Long Form of Fas Apoptotic Inhibitory Molecule Is Expressed Specifically in Neurons and Protects Them against Death Receptor-Triggered Apoptosis. J Neurosci [Internet]. 2007 10 17 [cited 2018 Jun 12];27(42):11228–41. Available from: http://www.ncbi.nlm.nih.gov/pubmed/17942717 10.1523/JNEUROSCI.3462-07.2007 17942717 PMC6673028

[34] Krasilnikov MA. Phosphatidylinositol-3 kinase dependent pathways: the role in control of cell growth, survival, and malignant transformation. Biochemistry (Mosc) [Internet]. 2000 1 [cited 2018 Jun 12];65(1):59–67. Available from: http://www.ncbi.nlm.nih.gov/pubmed/10702641 10702641

[35] Castaing-Berthou A, Malet N, Radojkovic C, Cabou C, Gayral S, Martinez LO, et al PI3Kβ Plays a Key Role in Apolipoprotein A-I-Induced Endothelial Cell Proliferation Through Activation of the Ecto-F1-ATPase/P2Y1 Receptors. Cell Physiol Biochem [Internet]. 2017 [cited 2018 Jun 11];42(2):579–93. Available from: http://www.ncbi.nlm.nih.gov/pubmed/28578353 10.1159/000477607 28578353

[36] Couarch P, Vernia S, Gourfinkel-An I, Lesca G, Gataullina S, Fedirko E, et al Lafora progressive myoclonus epilepsy: NHLRC1 mutations affect glycogen metabolism. J Mol Med (Berl) [Internet]. 2011 9 [cited 2018 Jun 11];89(9):915–25. Available from: http://www.ncbi.nlm.nih.gov/pubmed/21505799 21505799 10.1007/s00109-011-0758-y PMC3154284

[37] Giambartolomei C, Vukcevic D, Schadt EE, Franke L, Hingorani AD, Wallace C, et al Bayesian test for colocalisation between pairs of genetic association studies using summary statistics. PLoS Genet [Internet]. 2014 5 [cited 2019 Jul 5];10(5):e1004383 Available from: http://www.ncbi.nlm.nih.gov/pubmed/24830394 10.1371/journal.pgen.1004383 24830394 PMC4022491

[38] Võsa U, Claringbould A, Westra H-J, Bonder MJ, Deelen P, Zeng B, et al Unraveling the polygenic architecture of complex traits using blood eQTL metaanalysis. bioRxiv [Internet]. 2018 10 19 [cited 2019 Jul 5];447367. Available from: https://www.biorxiv.org/content/10.1101/447367v1

[39] de Leeuw CA, Mooij JM, Heskes T, Posthuma D. MAGMA: generalized gene-set analysis of GWAS data. PLoS Comput Biol [Internet]. 2015 4 [cited 2018 May 15];11(4):e1004219 Available from: http://www.ncbi.nlm.nih.gov/pubmed/25885710 10.1371/journal.pcbi.1004219 25885710 PMC4401657

[40] Krynetski EY, Evans WE. Genetic polymorphism of thiopurine S-methyltransferase: molecular mechanisms and clinical importance. Pharmacology [Internet]. 2000 9 [cited 2018 Jun 11];61(3):136–46. Available from: http://www.ncbi.nlm.nih.gov/pubmed/10971199 10.1159/000028394 10971199

[41] Nagaoka K, Hino S, Sakamoto A, Anan K, Takase R, Umehara T, et al Lysine-specific demethylase 2 suppresses lipid influx and metabolism in hepatic cells. Mol Cell Biol [Internet]. 2015 4 [cited 2018 Jun 11];35(7):1068–80. Available from: http://www.ncbi.nlm.nih.gov/pubmed/25624347 10.1128/MCB.01404-14 25624347 PMC4355535

[42] Ozato K, Shin D-M, Chang T-H, Morse HC. TRIM family proteins and their emerging roles in innate immunity. Nat Rev Immunol [Internet]. 2008 11 1 [cited 2018 Jun 11];8(11):849–60. Available from: http://www.nature.com/articles/nri2413 10.1038/nri2413 18836477 PMC3433745

[43] Yang J, Lu C, Wei J, Guo Y, Liu W, Luo L, et al Inhibition of KPNA4 attenuates prostate cancer metastasis. Oncogene [Internet]. 2017 [cited 2018 Jun 11];36(20):2868–78. Available from: http://www.ncbi.nlm.nih.gov/pubmed/27941876 10.1038/onc.2016.440 27941876 PMC5436935

[44] Jakob S, Haendeler J. Molecular mechanisms involved in endothelial cell aging: role of telomerase reverse transcriptase. Z Gerontol Geriatr [Internet]. 2007 10 [cited 2018 Jun 11];40(5):334–8. Available from: http://link.springer.com/10.1007/s00391-007-0482-y 17943236 10.1007/s00391-007-0482-y

[45] Mattson MP, Klapper W. Emerging roles for telomerase in neuronal development and apoptosis. J Neurosci Res [Internet]. 2001 1 1 [cited 2018 Jun 11];63(1):1–9. Available from: http://doi.wiley.com/10.1002/1097-4547%2820010101%2963%3A1%3C1%3A%3AAID-JNR1%3E3.0.CO%3B2-I 10.1002/1097-4547(20010101)63:1<1::AID-JNR1>3.0.CO;2-I 11169608

[46] Tajima H, Niikura T, Hashimoto Y, Ito Y, Kita Y, Terashita K, et al Evidence for in vivo production of Humanin peptide, a neuroprotective factor against Alzheimer’s disease-related insults. Neurosci Lett [Internet]. 2002 5 24 [cited 2018 Jun 11];324(3):227–31. Available from: https://www.sciencedirect.com/science/article/pii/S0304394002001994?via%3Dihub 10.1016/s0304-3940(02)00199-4 12009529

[47] Guo B, Zhai D, Cabezas E, Welsh K, Nouraini S, Satterthwait AC, et al Humanin peptide suppresses apoptosis by interfering with Bax activation. Nature [Internet]. 2003 5 4 [cited 2018 Jun 11];423(6938):456–61. Available from: http://www.nature.com/articles/nature01627 10.1038/nature01627 12732850

[48] Agrawal B, Krantz MJ, Parker J, Longenecker BM. Expression of MUC1 mucin on activated human T cells: implications for a role of MUC1 in normal immune regulation. Cancer Res [Internet]. 1998 9 15 [cited 2018 Jun 11];58(18):4079–81. Available from: http://www.ncbi.nlm.nih.gov/pubmed/9751614 9751614

[49] Shi Y, Yuan B, Zhu W, Zhang R, Li L, Hao X, et al Ube2D3 and Ube2N are essential for RIG-I-mediated MAVS aggregation in antiviral innate immunity. Nat Commun [Internet]. 2017 5 4 [cited 2018 Jun 12];8:15138 Available from: http://www.nature.com/doifinder/10.1038/ncomms15138 28469175 10.1038/ncomms15138 PMC5418627

[50] Chen Y-F, Wu C-Y, Kirby R, Kao C-H, Tsai T-F. A role for the CISD2 gene in lifespan control and human disease. Ann N Y Acad Sci [Internet]. 2010 7 [cited 2018 Jun 12];1201(1):58–64. Available from: http://doi.wiley.com/10.1111/j.1749-6632.2010.05619.x 20649540 10.1111/j.1749-6632.2010.05619.x

[51] Wang C-H, Kao C-H, Chen Y-F, Wei Y-H, Tsai T-F. Cisd2 mediates lifespan: is there an interconnection among Ca ^2+^ homeostasis, autophagy, and lifespan? Free Radic Res [Internet]. 2014 9 29 [cited 2018 Jun 12];48(9):1109–14. Available from: http://www.tandfonline.com/doi/full/10.3109/10715762.2014.936431 24974737 10.3109/10715762.2014.936431

[52] Bulik-Sullivan B, Finucane HK, Anttila V, Gusev A, Day FR, Loh P-R, et al An atlas of genetic correlations across human diseases and traits. Nat Genet [Internet]. 2015 11 28 [cited 2018 May 15];47(11):1236–41. Available from: http://www.nature.com/articles/ng.3406 10.1038/ng.3406 26414676 PMC4797329

[53] Zheng J, Erzurumluoglu AM, Elsworth BL, Kemp JP, Howe L, Haycock PC, et al LD Hub: a centralized database and web interface to perform LD score regression that maximizes the potential of summary level GWAS data for SNP heritability and genetic correlation analysis. Bioinformatics [Internet]. 2017 1 15 [cited 2018 May 15];33(2):272–9. Available from: https://academic.oup.com/bioinformatics/article-lookup/doi/10.1093/bioinformatics/btw613 27663502 10.1093/bioinformatics/btw613 PMC5542030

[54] Bell JT, Tsai P-C, Yang T-P, Pidsley R, Nisbet J, Glass D, et al Epigenome-wide scans identify differentially methylated regions for age and age-related phenotypes in a healthy ageing population. PLoS Genet [Internet]. 2012 [cited 2019 Jun 15];8(4):e1002629 Available from: http://www.ncbi.nlm.nih.gov/pubmed/22532803 10.1371/journal.pgen.1002629 22532803 PMC3330116

[55] Kops GJPL, Medema RH, Glassford J, Essers MAG, Dijkers PF, Coffer PJ, et al Control of cell cycle exit and entry by protein kinase B-regulated forkhead transcription factors. Mol Cell Biol [Internet]. 2002 4 [cited 2018 May 23];22(7):2025–36. Available from: http://www.ncbi.nlm.nih.gov/pubmed/11884591 10.1128/MCB.22.7.2025-2036.2002 11884591 PMC133681

[56] Bonafè M, Barbieri M, Marchegiani F, Olivieri F, Ragno E, Giampieri C, et al Polymorphic Variants of Insulin-Like Growth Factor I (IGF-I) Receptor and Phosphoinositide 3-Kinase Genes Affect IGF-I Plasma Levels and Human Longevity: Cues for an Evolutionarily Conserved Mechanism of Life Span Control. J Clin Endocrinol Metab [Internet]. 2003 7 1 [cited 2018 May 23];88(7):3299–304. Available from: https://academic.oup.com/jcem/article-lookup/doi/10.1210/jc.2002-021810 12843179 10.1210/jc.2002-021810

[57] Wu C-Y, Chen Y-F, Wang C-H, Kao C-H, Zhuang H-W, Chen C-C, et al A persistent level of Cisd2 extends healthy lifespan and delays aging in mice. Hum Mol Genet [Internet]. 2012 9 15 [cited 2018 May 23];21(18):3956–68. Available from: https://academic.oup.com/hmg/article-lookup/doi/10.1093/hmg/dds210 22661501 10.1093/hmg/dds210

[58] Chen Y-F, Kao C-H, Chen Y-T, Wang C-H, Wu C-Y, Tsai C-Y, et al Cisd2 deficiency drives premature aging and causes mitochondria-mediated defects in mice. Genes Dev [Internet]. 2009 5 15 [cited 2018 May 23];23(10):1183–94. Available from: http://www.ncbi.nlm.nih.gov/pubmed/19451219 10.1101/gad.1779509 19451219 PMC2685531

[59] Vågerö D, Aronsson V, Modin B. Why is parental lifespan linked to children’s chances of reaching a high age? A transgenerational hypothesis. SSM—Popul Heal [Internet]. 2018 4 [cited 2018 Nov 5];4:45–54. Available from: http://www.ncbi.nlm.nih.gov/pubmed/29349272 10.1016/j.ssmph.2017.11.006 PMC5769101 29349272

[60] Horvath S, Gurven M, Levine ME, Trumble BC, Kaplan H, Allayee H, et al An epigenetic clock analysis of race/ethnicity, sex, and coronary heart disease. Genome Biol [Internet]. 2016 12 11 [cited 2018 May 14];17(1):171 Available from: http://genomebiology.biomedcentral.com/articles/10.1186/s13059-016-1030-0 27511193 10.1186/s13059-016-1030-0 PMC4980791

[61] Smith BH, Campbell H, Blackwood D, Connell J, Connor M, Deary IJ, et al Generation Scotland: the Scottish Family Health Study; a new resource for researching genes and heritability. BMC Med Genet [Internet]. 2006 12 2 [cited 2018 May 15];7(1):74 Available from: http://bmcmedgenet.biomedcentral.com/articles/10.1186/1471-2350-7-74 17014726 10.1186/1471-2350-7-74 PMC1592477

[62] Smith BH, Campbell A, Linksted P, Fitzpatrick B, Jackson C, Kerr SM, et al Cohort Profile: Generation Scotland: Scottish Family Health Study (GS:SFHS). The study, its participants and their potential for genetic research on health and illness. Int J Epidemiol [Internet]. 2013 6 1 [cited 2018 May 15];42(3):689–700. Available from: https://academic.oup.com/ije/article-lookup/doi/10.1093/ije/dys084 22786799 10.1093/ije/dys084

[63] Houseman EA, Accomando WP, Koestler DC, Christensen BC, Marsit CJ, Nelson HH, et al DNA methylation arrays as surrogate measures of cell mixture distribution. BMC Bioinformatics [Internet]. 2012 5 8 [cited 2018 Jun 4];13:86 Available from: http://www.ncbi.nlm.nih.gov/pubmed/22568884 10.1186/1471-2105-13-86 22568884 PMC3532182

[64] Nagy R, Boutin TS, Marten J, Huffman JE, Kerr SM, Campbell A, et al Exploration of haplotype research consortium imputation for genome-wide association studies in 20,032 Generation Scotland participants. Genome Med [Internet]. 2017 12 7 [cited 2018 May 15];9(1):23 Available from: http://genomemedicine.biomedcentral.com/articles/10.1186/s13073-017-0414-4 28270201 10.1186/s13073-017-0414-4 PMC5339960

[65] Yang J, Zaitlen NA, Goddard ME, Visscher PM, Price AL. Advantages and pitfalls in the application of mixed-model association methods. Nat Genet [Internet]. 2014 2 1 [cited 2018 May 14];46(2):100–6. Available from: http://www.nature.com/articles/ng.2876 10.1038/ng.2876 24473328 PMC3989144

[66] Yang J, Lee SH, Goddard ME, Visscher PM. GCTA: A Tool for Genome-wide Complex Trait Analysis. Am J Hum Genet [Internet]. 2011 1 7 [cited 2018 May 14];88(1):76–82. Available from: https://www.sciencedirect.com/science/article/pii/S0002929710005987?via%3Dihub 10.1016/j.ajhg.2010.11.011 21167468 PMC3014363

[67] Zaitlen N, Kraft P, Patterson N, Pasaniuc B, Bhatia G, Pollack S, et al Using Extended Genealogy to Estimate Components of Heritability for 23 Quantitative and Dichotomous Traits. Visscher PM, editor. PLoS Genet [Internet]. 2013 5 30 [cited 2018 May 15];9(5):e1003520 Available from: http://dx.plos.org/10.1371/journal.pgen.1003520 23737753 10.1371/journal.pgen.1003520 PMC3667752

[68] Hall LS, Adams MJ, Arnau-Soler A, Clarke T-K, Howard DM, Zeng Y, et al Genome-wide meta-analyses of stratified depression in Generation Scotland and UK Biobank. Transl Psychiatry [Internet]. 2018 12; Available from: 10.1038/s41398-017-0034-1 PMC5802463 29317602

[69] Willer CJ, Li Y, Abecasis GR. METAL: fast and efficient meta-analysis of genomewide association scans. Bioinformatics [Internet]. 2010 9 1 [cited 2018 May 14];26(17):2190–1. Available from: http://www.ncbi.nlm.nih.gov/pubmed/20616382 10.1093/bioinformatics/btq340 20616382 PMC2922887

[70] Gibbs RA, Boerwinkle E, Doddapaneni H, Han Y, Korchina V, Kovar C, et al A global reference for human genetic variation. Nature [Internet]. 2015 10 1 [cited 2018 Sep 28];526(7571):68–74. Available from: http://www.nature.com/articles/nature15393 10.1038/nature15393 26432245 PMC4750478

[71] Welter D, MacArthur J, Morales J, Burdett T, Hall P, Junkins H, et al The NHGRI GWAS Catalog, a curated resource of SNP-trait associations. Nucleic Acids Res [Internet]. 2014 1 1 [cited 2018 May 15];42(D1):D1001–6. Available from: https://academic.oup.com/nar/article-lookup/doi/10.1093/nar/gkt1229 24316577 10.1093/nar/gkt1229 PMC3965119

[72] MacArthur J, Bowler E, Cerezo M, Gil L, Hall P, Hastings E, et al The new NHGRI-EBI Catalog of published genome-wide association studies (GWAS Catalog). Nucleic Acids Res [Internet]. 2017 1 4 [cited 2018 May 15];45(D1):D896–901. Available from: https://academic.oup.com/nar/article-lookup/doi/10.1093/nar/gkw1133 27899670 10.1093/nar/gkw1133 PMC5210590

[73] Consortium T 1000 GP. An integrated map of genetic variation from 1,092 human genomes. Nature [Internet]. 2012 11 [cited 2018 May 14];491(7422):56–65. Available from: http://www.nature.com/articles/nature11632 10.1038/nature11632 23128226 PMC3498066

[74] Wang K, Li M, Hakonarson H. ANNOVAR: functional annotation of genetic variants from high-throughput sequencing data. Nucleic Acids Res [Internet]. 2010 9 1 [cited 2018 May 14];38(16):e164–e164. Available from: https://academic.oup.com/nar/article-lookup/doi/10.1093/nar/gkq603 20601685 10.1093/nar/gkq603 PMC2938201

[75] Ernst J, Kellis M. ChromHMM: automating chromatin-state discovery and characterization. Nat Methods [Internet]. 2012 3 1 [cited 2018 May 14];9(3):215–6. Available from: http://www.nature.com/articles/nmeth.1906 10.1038/nmeth.1906 22373907 PMC3577932

[76] Kundaje A, Meuleman W, Ernst J, Bilenky M, Yen A, Heravi-Moussavi A, et al Integrative analysis of 111 reference human epigenomes. Nature [Internet]. 2015 2 19 [cited 2018 May 14];518(7539):317–30. Available from: http://www.nature.com/articles/nature14248 10.1038/nature14248 25693563 PMC4530010

[77] Zhu Z, Zhang F, Hu H, Bakshi A, Robinson MR, Powell JE, et al Integration of summary data from GWAS and eQTL studies predicts complex trait gene targets. Nat Genet [Internet]. 2016 5 28 [cited 2018 May 14];48(5):481–7. Available from: http://www.nature.com/articles/ng.3538 10.1038/ng.3538 27019110

[78] Benjamini Y, Hochberg Y. Controlling the False Discovery Rate: A Practical and Powerful Approach to Multiple Testing. Source J R Stat Soc Ser B [Internet]. 1995 [cited 2018 May 15];57(1):289–300. Available from: http://www.jstor.org/stable/2346101

