# Supplementary material for: A meta-analysis of genome-wide association studies of epigenetic age acceleration
Source: PLoS Genet. 2019 Nov 18;15(11):e1008104. doi: 10.1371/journal.pgen.1008104 (PMC6886870; doi:10.1371/journal.pgen.1008104)
Supplement: S1 Text — contains further information on the Hannum and Horvath epigenetic clocks, measures of epigenetic age and epigenetic age acceleration, DNA methylation in GS, derivation of epigenetic age and epigenetic age acceleration variables in GS, genotyping, imputation, and quality control in GS. (DOCX) [file pgen.1008104.s001.docx]

**The Hannum and Horvath epigenetic clocks**

The Hannum epigenetic clock was derived using DNA methylation profiles from whole blood in two cohorts comprising 656 individuals in total (1). This clock accurately estimates age based on blood methylation levels at 71 CpG sites from the Illumina 450K array (1). The Horvath epigenetic clock is based on methylation levels at 353 CpG sites common to both the Illumina 27K and 450K arrays (2). This was the first multi-tissue epigenetic clock, derived using DNA methylation data from 51 tissue types from almost 8,000 individuals across multiple studies (2). The major innovation of Horvath's epigenetic clock lies in its wide applicability – the same set of CpGs and the same prediction algorithm are used irrespective of the DNA source, with no adjustments required, so the ages of different tissues and organs from the same individual can be compared using the same ageing clock (2). In contrast, Hannum's blood-based age predictor is specific to blood samples; when applied to other tissues it leads to a high error due to poor calibration. It can, however, be adjusted for each tissue type using a linear model (1). Hannum's clock can not be used to compare the ages of different tissues or organs, but lends itself to comparing diseased (e.g. cancerous) tissue with normal tissue (1).

Although the selection of CpG sites for both the Hannum and Horvath predictors was done using a similar penalised regression model, they have only six CpG sites in common. Despite this, independent studies have reported moderate (r=0.37) (3) to fairly strong (r=0.76) (4) correlations between the clocks. The two DNA methylation age measures are clearly related, but capture slightly different aspects of biology (4). The Hannum age estimator is correlated with proportions of certain blood cells, reflecting that it was constructed on the basis of whole blood DNA methylation data (4,5). It is considered to track aspects of immunosenescence, having been found to exhibit significant, though weak, correlations with several markers of immunosenescence such as abundance of senescent T cells and telomere length (5,6). Horvath's clock is relatively uncorrelated with blood cell counts (7), reflecting that it was constructed across a broad spectrum of tissues and cell types. It does not relate to measures of immunosenescence such as telomere length (6), and is considered to instead capture cell intrinsic changes in DNA methylation age which might reflect an innate ageing process.

**Measures of epigenetic age and epigenetic age acceleration**

Despite high correlations overall, epigenetic age can deviate substantially from chronological age at the individual level. “Epigenetic age acceleration” (EAA) is defined as the residual that results from regressing epigenetic age on chronological age. Thus positive values of EAA indicate that the epigenetic age is higher than expected, based on chronological age.

Owing to the well documented changes to blood cell composition with age (8,9), two broad categories of epigenetic ageing measures have been distinguished: those that are independent of age-related changes in blood cell composition, and those that incorporate and are enhanced by blood cell count information (4). The former group, considered to reflect ‘pure’ epigenetic ageing effects that are not influenced by differences in blood cell counts, are often referred to as ‘intrinsic’ epigenetic age measures, and are thought to capture cell-intrinsic properties of the ageing process that exhibit some preservation across various cell types and organs (4). The latter group up-weights the contributions of blood cell counts, thus leveraging known age-related changes to blood cell proportions to capture aspects of immunosenescence. These measures are referred to as ‘extrinsic’ epigenetic age measures, and reflect both cell intrinsic methylation changes and extracellular changes in blood cell composition (4).

**DNA methylation in Generation Scotland: Scottish Family Health Study**

At the time of this study, DNA methylation data were available for 5,200 individuals from Generation Scotland (GS). Whole blood genomic DNA samples were treated with sodium bisulphite using the EZ-96 DNA Methylation Kit (Zymo Research, Irvine, California), following the manufacturer's instructions. Genome-wide DNA methylation was measured using the Infinium MethylationEPIC BeadChip (Illumina Inc., San Diego, California) in accordance with the manufacturer's protocol. Arrays were scanned using a HiScan scanner (Illumina Inc., San Diego, California), with initial inspection of array quality carried out in GenomeStudio v2011.1. Additional quality control measures were implemented using the R packages 'shinyMethyl' (10) and 'wateRmelon' (11). ShinyMethyl was used to plot the log median intensity of methylated versus unmethylated signal for each array, with outliers identified upon visual inspection and excluded. Methylation beta-values were then entered into the 'pfilter' function in wateRmelon, which was used to exclude poorly performing samples and probes, removing samples where ≥1% of CpGs had a detection *P*-value in excess of 0.05, and probes for which more than five samples had a beadcount of less than three or for which ≥0.5% of samples had a detection *P*-value in excess of 0.05. Finally, shinyMethyl's sex prediction plot was used to exclude samples for which predicted sex differed from their recorded sex. After this QC, the dataset comprised beta-values for 860,928 methylation loci measured in 5,101 individuals. The DNA methylation data were used to derive estimates ‘epigenetic age’ and ‘epigenetic age acceleration’.

**Derivation of epigenetic age and epigenetic age acceleration variables in GS**

In keeping with previous work, this study derived two different epigenetic age estimates for each sample, based on the Horvath and Hannum epigenetic clocks (1,2). Horvath-based epigenetic age followed the approach by Horvath (2013), and is defined as the predicted value of age based on the DNA methylation levels of the 353 CpG sites identified in his study (2). Hannum-based epigenetic age is based on DNA methylation levels at the 71 CpGs identified by Hannum et al. (2013) (1). Using the Horvath-based and Hannum-based epigenetic age estimates, we derived variations of EAA that are either independent of blood cell counts, or enhanced by changes in blood cell composition.

Horvath-based epigenetic age acceleration (Horvath-EAA) is the residual term of a multivariate model regressing the Horvath-based epigenetic age estimate on chronological age and estimates of blood cell counts (naive CD8+ T cells, exhausted CD8+ T cells, plasmablasts, CD4+ T cells, natural killer cells, monocytes, and granulocytes) imputed from the methylation data. Horvath-EAA is by definition independent of both chronological age and variation in age-related changes in blood immune cell counts. This measure has been described in previous publications as intrinsic epigenetic age acceleration (IEAA), as it is independent of changes in the abundance of particular cell types in the blood.

Hannum-based epigenetic age acceleration (Hannum-EAA) is an enhanced version of the Hannum estimate which up-weights the contributions of age-associated blood cells. Hannum-based epigenetic age already correlates with certain blood cell types (5). The contribution of immune blood cell types to the age estimate can be increased by forming a weighted average (where the weights used are determined by the correlation between the respective variable and chronological age) of Hannum-based epigenetic age with three blood cell types whose abundance is known to change with age (naïve cytotoxic T-cells, exhausted cytotoxic T-cells, and plasmablasts) using the Klemera-Doubal approach (12). Hannum-EAA is then defined to be the residual variation from a univariate model regressing the weighted DNA methylation age estimate on chronological age, yielding an estimate which more strongly reflects ageing of the immune system (4,6,13). This measure has previously been described as extrinsic epigenetic age acceleration (EEAA), and tracks both cell-intrinsic epigenetic changes and age-related changes in blood cell composition.

For the current study, the Horvath-based and Hannum-based age estimates, and the two epigenetic age acceleration variables (Horvath-EAA and Hannum-EAA) were obtained from the freely available online DNA Methylation Age Calculator (<https://dnamage.genetics.ucla.edu/>) developed by Horvath (2). Normalised DNA methylation beta-values for 5,101 GS participants were submitted to the calculator, using the 'Advanced Analysis for Blood Data' option, and undergoing further normalisation within the calculator algorithm, to make the data comparable to the training data of the epigenetic clock. Horvath-EAA and Hannum-EAA, calculated as outlined above, were denoted as AAHOAdjCellCounts and BioAge4HAStaticAdjAge, respectively. Full mathematical details of the calculation methods can be found in the original publication (2). Blood cell abundance measures were also estimated by the online calculator, based on DNA methylation levels and using Houseman's estimation method, as described previously (14).

**Genotyping, imputation, and quality control in GS**

Blood or saliva samples for GS participants were collected, processed, and stored following standard operating procedures; full details of sample collection and DNA extraction are described elsewhere (15). Genotyping was performed using the Illumina HumanOmniExpressExome-8 v 1.0 DNA Analysis BeadChip and Infinium chemistry, with genotypes processed in GenomeStudio v2011.1, as described previously (16). Quality control procedures on the raw genotypes included removal of individuals with a call rate less than 98%, and SNPs with a call rate less than 99% or showing significant deviation from Hardy-Weinberg equilibrium (HWE *P*-value ≤1x10^−6^); full details of QC can be found elsewhere (17). A total of 20,032 individuals, including all participants for whom methylation data were also available, and 604,858 genotyped autosomal SNPs passed all quality control thresholds.

To increase the density of variants throughout the genome, genotypes were imputed using the Haplotype Research Consortium reference panel v1.1 (18) via the Sanger Imputation Server Pipeline ([https://imputation.sanger.ac.uk](https://imputation.sanger.ac.uk/)), as described previously (17). Phasing of genotype data was performed using the SHAPEIT2 algorithm (19) utilizing the duoHMM option, which refines phasing by utilizing pedigree information. Imputation was performed using PBWT software (20). Monomorphic or multi-allelic variants, and SNPs with a low imputation quality (INFO<0.4) were removed from the imputed dataset, leaving 24,111,857 variants available for downstream analysis. Given the relatively small sample size of the current study, only data from variants with a minor allele frequency greater than 1% were considered, resulting in an imputed dataset with 8,633,288 variants to be used in the genome-wide association analysis.

**References**

1. Hannum G, Guinney J, Zhao L, Zhang L, Hughes G, Sadda S, et al. Genome-wide methylation profiles reveal quantitative views of human aging rates. Mol Cell [Internet]. 2013 Jan 24 [cited 2018 May 14];49(2):359–67. Available from: http://www.ncbi.nlm.nih.gov/pubmed/23177740

2. Horvath S. DNA methylation age of human tissues and cell types. Genome Biol [Internet]. 2013 Dec 10 [cited 2018 May 14];14(10):R115. Available from: http://genomebiology.biomedcentral.com/articles/10.1186/gb-2013-14-10-r115

3. Belsky DW, Moffitt TE, Cohen AA, Corcoran DL, Levine ME, Prinz JA, et al. Eleven Telomere, Epigenetic Clock, and Biomarker-Composite Quantifications of Biological Aging: Do They Measure the Same Thing? Am J Epidemiol [Internet]. 2017 Nov 15 [cited 2019 Jan 21];187(6):1220–30. Available from: http://academic.oup.com/aje/article/doi/10.1093/aje/kwx346/4622080

4. Chen BH, Marioni RE, Colicino E, Peters MJ, Ward-Caviness CK, Tsai P-C, et al. DNA methylation-based measures of biological age: meta-analysis predicting time to death. Aging (Albany NY) [Internet]. 2016 Sep 28 [cited 2018 May 14];8(9):1844–65. Available from: http://www.ncbi.nlm.nih.gov/pubmed/27690265

5. Marioni RE, Shah S, McRae AF, Chen BH, Colicino E, Harris SE, et al. DNA methylation age of blood predicts all-cause mortality in later life. Genome Biol [Internet]. 2015 Jan 30 [cited 2018 May 14];16(1):25. Available from: http://genomebiology.com/2015/16/1/25

6. Chen BH, Carty CL, Kimura M, Kark JD, Chen W, Li S, et al. Leukocyte telomere length, T cell composition and DNA methylation age. Aging (Albany NY) [Internet]. 2017 [cited 2019 Jan 21];9(9):1983–95. Available from: http://www.ncbi.nlm.nih.gov/pubmed/28930701

7. Marioni RE, Suderman M, Chen BH, Horvath S, Bandinelli S, Morris T, et al. Tracking the Epigenetic Clock Across the Human Life Course: A Meta-analysis of Longitudinal Cohort Data. J Gerontol A Biol Sci Med Sci [Internet]. 2019 Jan 1 [cited 2019 Jan 21];74(1):57–61. Available from: http://www.ncbi.nlm.nih.gov/pubmed/29718110

8. Fagnoni FF, Vescovini R, Passeri G, Bologna G, Pedrazzoni M, Lavagetto G, et al. Shortage of circulating naive CD8(+) T cells provides new insights on immunodeficiency in aging. Blood [Internet]. 2000 May 1 [cited 2019 Jan 21];95(9):2860–8. Available from: http://www.ncbi.nlm.nih.gov/pubmed/10779432

9. Miller RA. The aging immune system: primer and prospectus. Science [Internet]. 1996 Jul 5 [cited 2019 Jan 21];273(5271):70–4. Available from: http://www.ncbi.nlm.nih.gov/pubmed/8658199

10. Fortin J-P, Fertig E, Hansen K. shinyMethyl: interactive quality control of Illumina 450k DNA methylation arrays in R. F1000Research [Internet]. 2014 [cited 2018 May 15];3:175. Available from: http://www.ncbi.nlm.nih.gov/pubmed/25285208

11. Pidsley R, Y Wong CC, Volta M, Lunnon K, Mill J, Schalkwyk LC. A data-driven approach to preprocessing Illumina 450K methylation array data. BMC Genomics [Internet]. 2013 May 1 [cited 2018 May 15];14(1):293. Available from: http://bmcgenomics.biomedcentral.com/articles/10.1186/1471-2164-14-293

12. Klemera P, Doubal S. A new approach to the concept and computation of biological age. Mech Ageing Dev [Internet]. 2006 Mar 1 [cited 2018 May 14];127(3):240–8. Available from: https://www.sciencedirect.com/science/article/pii/S0047637405002654?via%3Dihub

13. Quach A, Levine ME, Tanaka T, Lu AT, Chen BH, Ferrucci L, et al. Epigenetic clock analysis of diet, exercise, education, and lifestyle factors. Aging (Albany NY) [Internet]. 2017 Feb 14 [cited 2018 May 14];9(2):419–46. Available from: http://www.ncbi.nlm.nih.gov/pubmed/28198702

14. Houseman EA, Accomando WP, Koestler DC, Christensen BC, Marsit CJ, Nelson HH, et al. DNA methylation arrays as surrogate measures of cell mixture distribution. BMC Bioinformatics [Internet]. 2012 May 8 [cited 2018 Jun 4];13:86. Available from: http://www.ncbi.nlm.nih.gov/pubmed/22568884

15. Kerr SM, Campbell A, Murphy L, Hayward C, Jackson C, Wain L V, et al. Pedigree and genotyping quality analyses of over 10,000 DNA samples from the Generation Scotland: Scottish Family Health Study. BMC Med Genet [Internet]. 2013 Dec 22 [cited 2018 May 15];14(1):38. Available from: http://bmcmedgenet.biomedcentral.com/articles/10.1186/1471-2350-14-38

16. Gunderson KL. Whole-Genome Genotyping on Bead Arrays. In Humana Press; 2009 [cited 2018 May 15]. p. 197–213. Available from: http://link.springer.com/10.1007/978-1-59745-538-1_13

17. Nagy R, Boutin TS, Marten J, Huffman JE, Kerr SM, Campbell A, et al. Exploration of haplotype research consortium imputation for genome-wide association studies in 20,032 Generation Scotland participants. Genome Med [Internet]. 2017 Dec 7 [cited 2018 May 15];9(1):23. Available from: http://genomemedicine.biomedcentral.com/articles/10.1186/s13073-017-0414-4

18. Consortium the HR, McCarthy S, Das S, Kretzschmar W, Delaneau O, Wood AR, et al. A reference panel of 64,976 haplotypes for genotype imputation. Nat Genet [Internet]. 2016 Oct 22 [cited 2018 May 15];48(10):1279–83. Available from: http://www.nature.com/articles/ng.3643

19. Delaneau O, Marchini J, Zagury J-F. A linear complexity phasing method for thousands of genomes. Nat Methods [Internet]. 2012 Feb 4 [cited 2018 May 15];9(2):179–81. Available from: http://www.nature.com/articles/nmeth.1785

20. Durbin R. Efficient haplotype matching and storage using the positional Burrows-Wheeler transform (PBWT). Bioinformatics [Internet]. 2014 May 1 [cited 2018 May 14];30(9):1266–72. Available from: https://academic.oup.com/bioinformatics/article-lookup/doi/10.1093/bioinformatics/btu014
