# Supplementary material for: A meta-analysis of genome-wide association studies of epigenetic age acceleration
Source: PLoS Genet. 2019 Nov 18;15(11):e1008104. doi: 10.1371/journal.pgen.1008104 (PMC6886870; doi:10.1371/journal.pgen.1008104)
Supplement: S1 Fig — (DOCX) [file pgen.1008104.s021.docx]

**S1 Figure**: QQ plots for the GWAS of Horvath-EAA and Hannum-EAA in GS, showing the expected distribution of GWAS test statistics, -log10(p), versus the observed distribution.


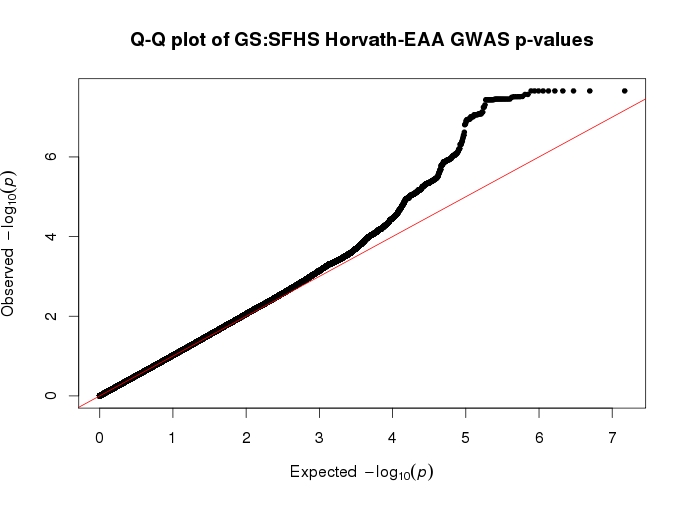

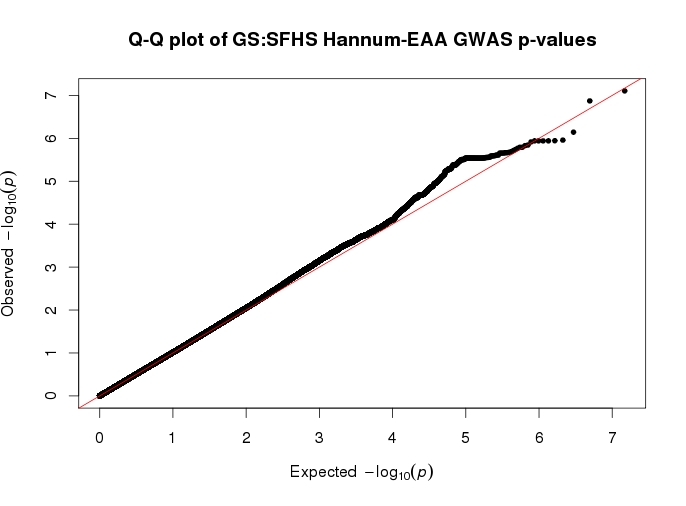


Univariate LD score regression using the GWAS summary statistics for GS gives a λ_GC_ estimate of 1.0165, and a LD score regression intercept of 1.0015 (0.0066) for Horvath-EAA, and λ_GC_ of 1.0225 and intercept of 0.9984 (0.0062) for Hannum-EAA.
