# Supplementary material for: A meta-analysis of genome-wide association studies of epigenetic age acceleration
Source: PLoS Genet. 2019 Nov 18;15(11):e1008104. doi: 10.1371/journal.pgen.1008104 (PMC6886870; doi:10.1371/journal.pgen.1008104)
Supplement: S2 Fig — Regional association plots for nine independent significantly associated SNPs for Horvath-EAA (A-I) and the single independent significantly associated SNP for Hannum-EAA (J), showing LD with SNPs in the surrounding region. Plots were produced in LocusZoom. The SNP association P-value is given on the y-axis, and SNP position, with gene annotation, on the x-axis. LD calculations are taken from hg19/1000 Genomes European build. Individual SNPs are coloured according to the strength of LD (r2) with the lead SNP. The highest association signal in each panel, highlighted in violet, are as follows: A: rs1011267, an intronic SNP in C1orf112 on chromosome 1; B: rs79070372, a non-coding transcript variant on chromosome 3 (closest genes GATA2/AS1); C: rs388649, an intronic SNP in PIK3CB on chromosome 3; D: rs6440667, an intronic SNP in LINC01214 on chromosome 3; E: rs2736099, an intronic SNP in TERT on chromosome 5; F: rs76244256, an intron variant in TPMT on chromosome 6 and the top ranking SNP for association with Horvath-EAA *This genomic locus contains a second independent associated SNP, intergenic variant rs7744541 (nearest gene NHLRC1), which remained significantly associated (P<5x10^-8^) with Horvath-EAA after conditioning on the lead SNP; G: rs4712953, an intronic SNP in SCGN on chromosome 6; H: rs10778517, a SNP of unknown function on chromosome 12 (nearest genes RP11-412D9.4 and TMEM263); I: rs62078811, an intron variant in STXBP4 on chromosome 17; J: rs1005277, the single independent Hannum-EAA significant associated SNP, a SNP of unknown function on chromosome 10 (nearest gene ZNF25). (DOCX) [file pgen.1008104.s022.docx]

**S2 Figure A-J**: Regional association plots for the independent SNPs that are significantly associated with Horvath-EAA. Plots were produced in LocusZoom. The SNP association *P*-value is given on the y-axis, and SNP position, with gene annotation, on the x-axis. LD calculations are taken from hg19/1000 Genomes European build. Individual SNPs are coloured according to the strength of LD (*r^2^*) with the lead SNP.

**S2A Figure**: Regional association plot for rs1011267, an intronic SNP in C1orf112 on chromosome 1, showing LD with SNPs in the surrounding region.

**S2B Figure**: Regional association plot for rs79070372, a non-coding transcript variant on chromosome 3 (closest genes GATA2/AS1), showing LD with SNPs in the surrounding region.

**S2C Figure**: Regional association plot for rs388649, an intronic SNP in PIK3CB on chromosome 3, showing LD with SNPs in the surrounding region.

**S2D Figure**: Regional association plot for rs6440667, an intronic SNP in LINC01214 on chromosome 3, showing LD with SNPs in the surrounding region.

**S2E Figure**: Regional association plot for rs2736099, an intronic SNP in TERT on chromosome 5, showing LD with SNPs in the surrounding region.

**S2F Figure**: Regional association plot for rs7744541, an intergenic variant on chromosome 6 (nearest gene NHLRC1), showing LD with SNPs in the surrounding region.

**S2F Figure**: Regional association plot for rs76244256, an intron variant in TPMT on chromosome 6 and the top ranking SNP for association with Horvath-EAA, showing LD with SNPs in the surrounding region.

**S2G Figure**: Regional association plot for rs4712953, an intronic SNP in SCGN on chromosome 6, showing LD with SNPs in the surrounding region.

**S2H Figure**: Regional association plot for rs10778517, a SNP of unknown function on chromosome 12 (nearest genes RP11-412D9.4 and TMEM263), showing LD with SNPs in the surrounding region.

**S2I Figure**: Regional association plot for rs62078811, an intron variant in STXBP4 on chromosome 17, showing LD with SNPs in the surrounding region.

**S2J Figure**: Regional association plot for the independent significantly associated SNP with Hannum-EAA, rs1005277. The plot was produced in LocusZoom. The SNP association *P-*value is given on the y-axis, and SNP position, with gene annotation, on the x-axis. LD calculations are taken from hg19/1000 Genomes European build. Individual SNPs are coloured according to the strength of LD (*r^2^*) with the lead SNP. The nearest gene to this SNP is the zinc finger protein ZNF25.
