# Supplementary material for: A meta-analysis of genome-wide association studies of epigenetic age acceleration
Source: PLoS Genet. 2019 Nov 18;15(11):e1008104. doi: 10.1371/journal.pgen.1008104 (PMC6886870; doi:10.1371/journal.pgen.1008104)

**S3 Figure**: Manhattan plots for the MAGMA gene-based association analysis for the GWAS meta-analysis (n=13,493) of Horvath-based epigenetic age acceleration and Hannum-based epigenetic age acceleration, with - log_10_ transformed *P*-values for each gene plotted against chromosomal location. The dotted line denotes genome-wide significance, defined at *P*=0.05/17798=2.809x10^-6^. Genes whose *P*-value reached genome-wide significance are labelled on the plots.


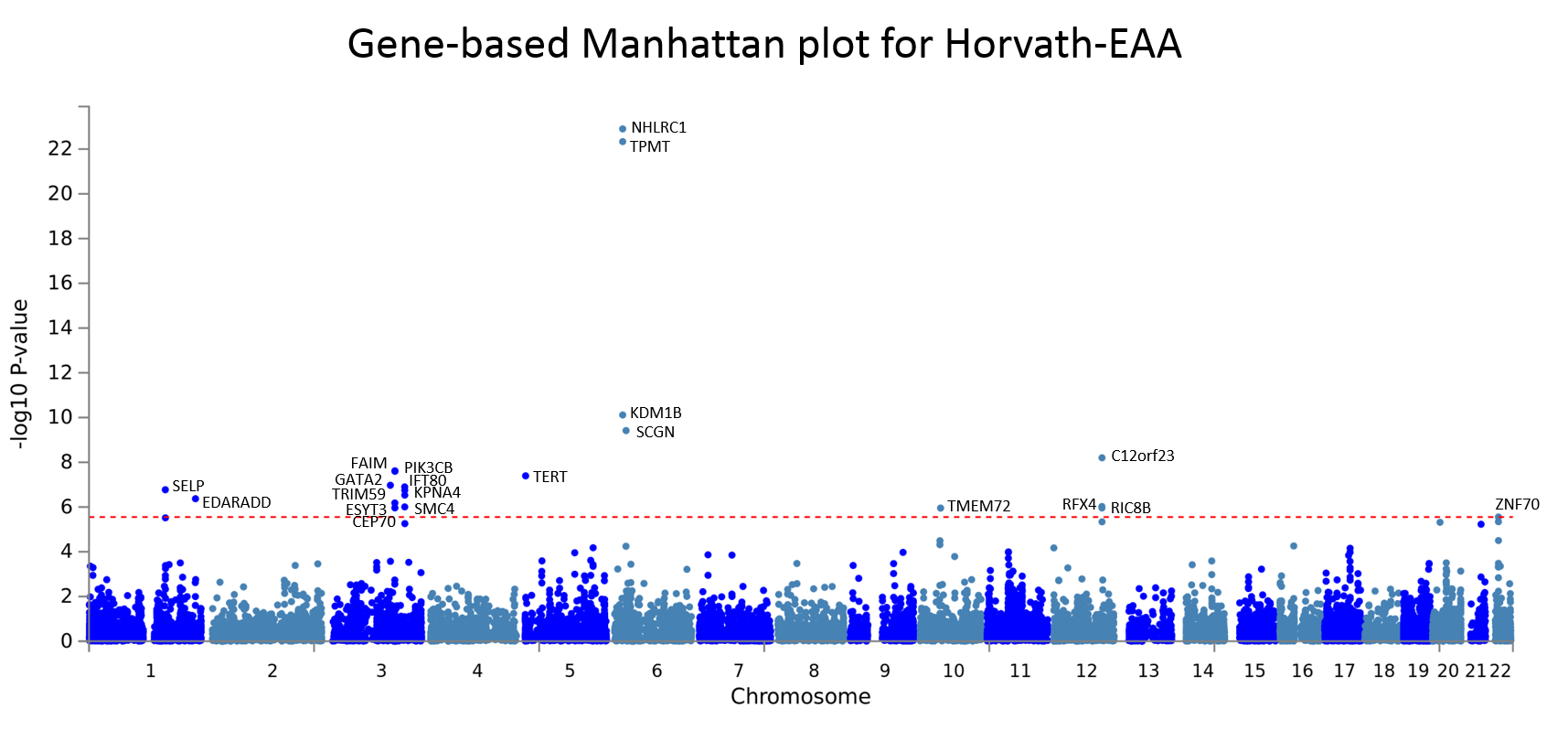


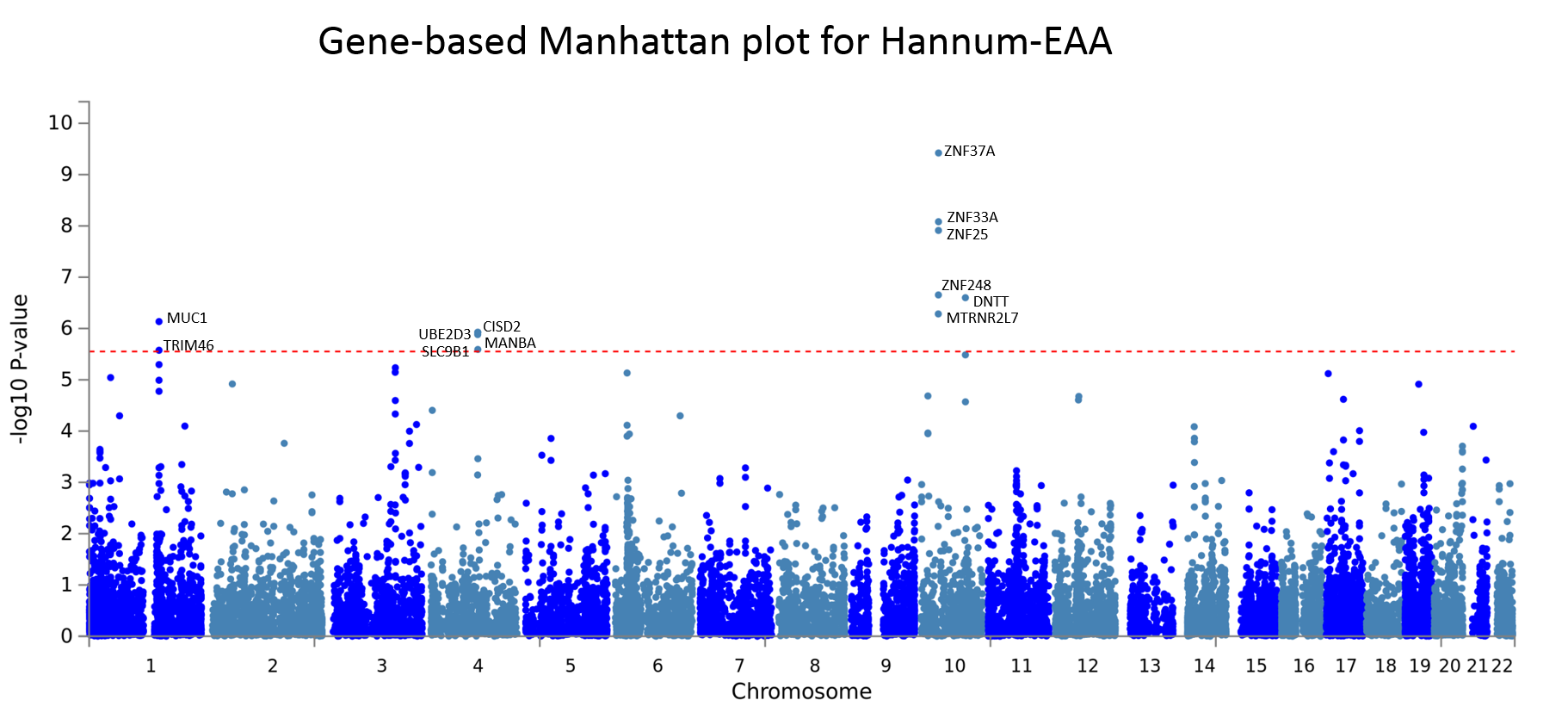

Supplement: S3 Fig — The dotted line denotes genome-wide significance, defined at P = 0.05/17798 = 2.809x10^-6^. Genes whose P-value reached genome-wide significance are labelled on the plots. (DOCX) [file pgen.1008104.s023.docx]
