# Supplementary material for: A meta-analysis of genome-wide association studies of epigenetic age acceleration
Source: PLoS Genet. 2019 Nov 18;15(11):e1008104. doi: 10.1371/journal.pgen.1008104 (PMC6886870; doi:10.1371/journal.pgen.1008104)

**S4 Figure**: QQ plots for the gene-based association analyses of Horvath-EAA and Hannum-EAA, showing the expected distribution of test statistics, -log10(p), versus the observed distribution.

QQ plot of gene-based test for Horvath-EAA QQ plot of gene-based test for Hannum-EAA


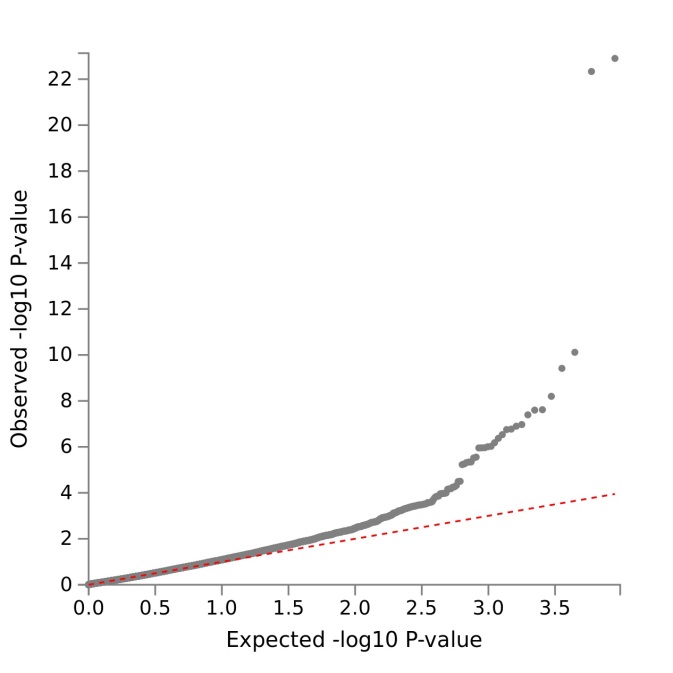

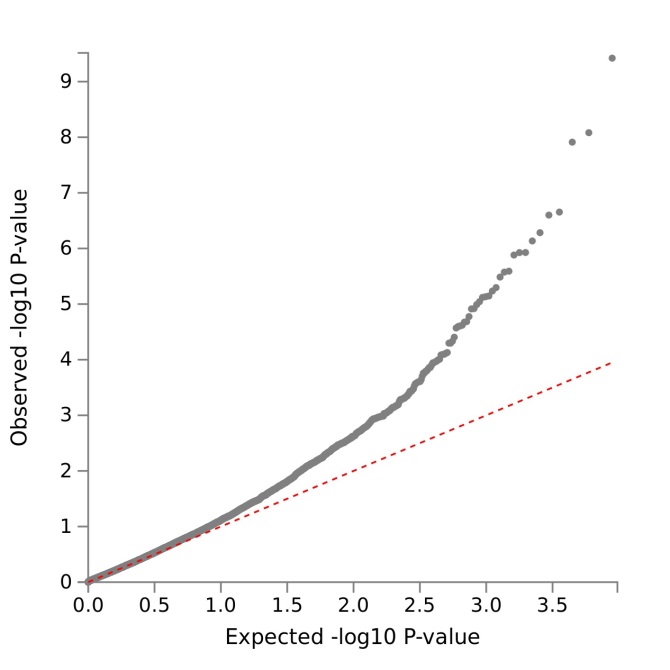

Supplement: S4 Fig — (DOCX) [file pgen.1008104.s024.docx]
